# Lineage recording in human cerebral organoids

**DOI:** 10.1038/s41592-021-01344-8

**Published:** 2021-12-30

**Authors:** Zhisong He, Ashley Maynard, Akanksha Jain, Tobias Gerber, Rebecca Petri, Hsiu-Chuan Lin, Malgorzata Santel, Kevin Ly, Jean-Samuel Dupré, Leila Sidow, Fatima Sanchis Calleja, Sophie M. J. Jansen, Stephan Riesenberg, J. Gray Camp, Barbara Treutlein

**Affiliations:** 1 Department of Biosystems Science and Engineering, ETH Zürich, Basel, Switzerland; 2 Max Planck Institute for Evolutionary Anthropology, Leipzig, Germany; 3 Institute of Molecular and Clinical Ophthalmology, Basel, Switzerland; 4 Department of Ophthalmology, University of Basel, Basel, Switzerland

**Keywords:** Neurogenesis, RNA sequencing, Gene expression, Stem-cell differentiation

## Abstract

Induced pluripotent stem cell (iPSC)-derived organoids provide models to study human organ development. Single-cell transcriptomics enable highly resolved descriptions of cell states within these systems; however, approaches are needed to directly measure lineage relationships. Here we establish iTracer, a lineage recorder that combines reporter barcodes with inducible CRISPR–Cas9 scarring and is compatible with single-cell and spatial transcriptomics. We apply iTracer to explore clonality and lineage dynamics during cerebral organoid development and identify a time window of fate restriction as well as variation in neurogenic dynamics between progenitor neuron families. We also establish long-term four-dimensional light-sheet microscopy for spatial lineage recording in cerebral organoids and confirm regional clonality in the developing neuroepithelium. We incorporate gene perturbation (iTracer-perturb) and assess the effect of mosaic *TSC2* mutations on cerebral organoid development. Our data shed light on how lineages and fates are established during cerebral organoid formation. More broadly, our techniques can be adapted in any iPSC-derived culture system to dissect lineage alterations during normal or perturbed development.

## Abstract (web summary)

A dual-channel recording system for high-resolution lineage tracing.

## Main

Three-dimensional (3D) tissues derived from human iPSCs (so-called organoids) mimic aspects of in vivo architecture and multilineage differentiation observed in primary developing tissues^1^. Single-cell RNA sequencing (scRNA-seq) has been extensively used to identify molecularly distinct cell types in organoids^2–7^ and assess organoid fidelity through comparisons with primary tissues^8^. scRNA-seq can also identify cells as intermediates between types, and cells can then be computationally aligned to delineate differentiation paths and order cells in pseudotime^9,10^. Methods such as RNA velocity provide an additional layer of information to assist with inferring differentiation trajectory and fate potentials^11^. However, it is not possible to use these inferences to identify direct cell lineages; therefore, it remains difficult to study how lineages are established during organoid self-organization. Several lineage-coupled single-cell transcriptomic strategies have emerged to investigate clonal expansion and differentiation in mouse and zebrafish embryos as well as complex multicellular culture systems^12–16^. These efforts rely on either expression of reporter transcripts tagged with a unique sequence barcode^12,16^, scarring patterns generated by CRISPR–Cas9 genomic modification of expressed targets^13–15^ or a combination of the two^15,17^. Lineage-coupled scRNA-seq has allowed for better annotation of cell fate specifications and trajectory inferences in complex tissues and other cell differentiation scenarios. Additionally, image-based methods such as four-dimensional (4D) light-sheet microscopy provide a complementary approach to capture comprehensive developmental dynamics in tissues and embryos^18,19^. Long-term live 4D light-sheet microscopy of a sample with minimum phototoxicity over days allows for observation and quantification of dynamic cellular behaviors at high spatiotemporal resolution^20,21^. Subsequent analysis allows for visualization, annotation and accurate lineage reconstruction of development. Lineage-coupled single-cell transcriptomics and long-term light-sheet microscopy therefore offer complementary approaches to record and understand lineage dynamics in iPSC-derived organoid systems.

Here we establish a dual-channel cell lineage recorder that allows clone tracing from an initializing iPSC pool while also enabling lineage recording at distinct time points using an inducible scar. This system (called iTracer) enables both clonal analysis as well as exploration of the temporal dynamics of cell fate establishment, avoiding multiple rounds of labeling. We use iTracer to understand lineage dynamics during cerebral organoid^22^ brain regionalization. Immunohistochemistry has shown that diverse brain regions can form within these tissues, and the relative positions of certain brain regions suggest that regional patterning gradients emerge that are reflective of in vivo development^23^. It was shown in forebrain organoid models that the range of dorsoventral identities can be generated within a continuous neuroepithelium and that organizing centers emerge that express secreted growth factors associated with dorsoventral patterning^22^. Single-cell transcriptome analysis over a time course of cerebral organoid development confirmed that diverse brain regions form within individual organoids^5^ and that brain region signatures in organoids strongly resemble counterparts from spatial in situ atlases of the developing mouse brain^24^. However, very little is known about spatiotemporal lineage dynamics during organoid formation and patterning or how these dynamics relate to the establishment of neuronal fates.

We used iTracer to explore lineages coupled with molecular signatures during brain organoid patterning and neurogenesis and show that the system is compatible with spatial transcriptomics. We also leverage 4D light-sheet microscopy to provide an assessment of clonal and morphological dynamics as the neuroepithelium develops at the onset of brain regionalization. We incorporate CRISPR-based gene perturbation into the lineage-recorder system (iTracer-perturb) to enable temporal control of gene loss of function during organoid development, providing both transcriptomic and cell lineage readouts. In proof-of-principle experiments, we target *TSC2* (encoding tuberous sclerosis complex 2), a gene associated with neurodevelopmental dysplasias^25–27^. Using these methodologies, we suggest that clone restriction plays a fundamental role in cerebral organoid regionalization, identify patterning profiles that are consistent with in vivo brain development and find that *TSC2* gene perturbations may augment metabolism and delay neural development.

## Results

### iTracer: a dynamic dual-channel lineage recorder

**Fig. 1:**
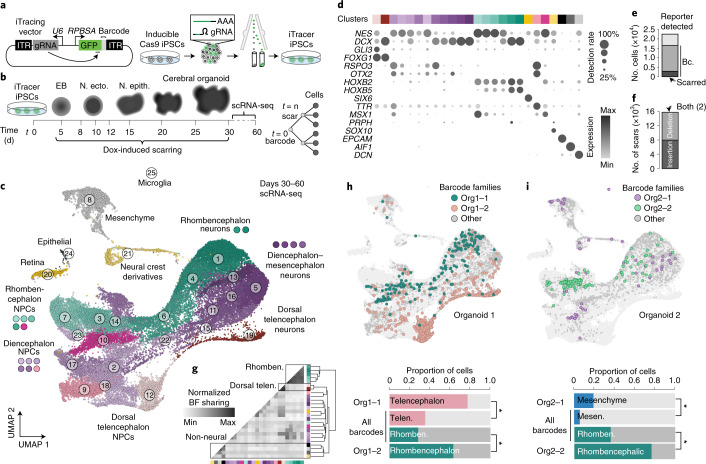
iTracer dual-channel lineage recorder uncovers clonality of cell fates in human cerebral organoids. **a**, Schematic of the iTracer Sleeping Beauty vector used for lineage recording. ITR, inverted terminal repeat. **b**, Scarring is induced at different time points of organoid development through doxycycline (dox) induction of Cas9. N. ecto., neuroectoderm; N. epith., neuroepithelial. **c**, UMAP embedding of scRNA-seq data of 44,275 cells from 12 cerebral organoids after data integration using CSS^16^. Cells are colored and numbered by transcriptome cluster and labeled with brain region and cell type annotations. **d**, Dot plot shows expression of genes marking clusters observed in organoids. **e**, Stacked bar plot showing the number of cells in which the iTracer reporter was detected (light gray), with only barcodes (Bc., dark gray) or barcodes and scars (black). Max, maximum; min, minimum. **f**, Stacked bar plot showing the number of scars created from insertions (light gray), deletions (dark gray) or both. **g**, Heatmap showing relative proportion of shared barcodes between transcriptome clusters. Rhomben., rhombencephalon; telen., telencephalon. **h**,**i**, Top, UMAP embedding as in **c**, colored by four different clones, showing that iPSC clones tend to accumulate in distinct brain regions or cell types. Bottom, bar plots showing proportions of cells of specific barcode families or all barcoded cells annotated with a given regional identity. Mesen., mesenchyme. Fisher’s exact test (two sided) was performed, comparing cell frequencies of a barcode family with those of all barcoded cells in the organoid in different clusters; *P < 0.0001.

**Extended Data Fig. 1:**
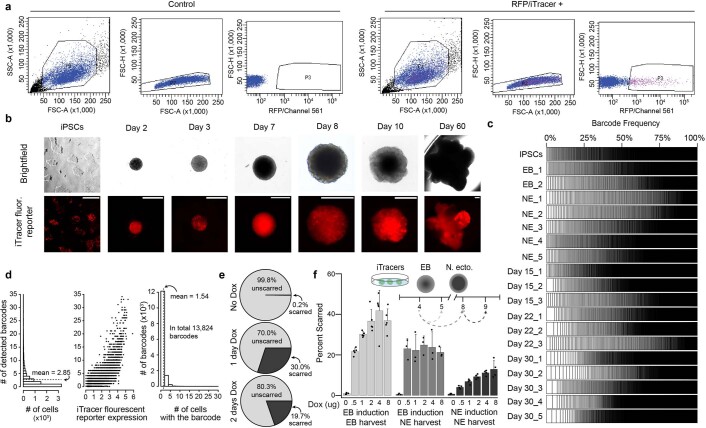
Assessment of iTracer readouts iPSC and iPSC-derived cerebral organoids. (**a**) FACs plots of sorting scheme used to isolate iTracer positive cells. Cells were gated first based on the main population to avoid debris, followed by gating for single cells excluding doublets, and lastly sorted for RFP or GFP positive cells as compared to non-fluorescent negative controls. (**b**) Brightfield and fluorescence imaging of iTracer reporter (RFP) in representative organoids during cerebral organoid development. Scale bar is 500 µm in all images (**c**) Stacked bar plots showing frequency of unique barcodes detected in bulk targeted amplicon sequencing libraries throughout cerebral organoid development. (**d**) Barplots of the number of iTracer barcodes detected from single-cell transcriptomes. The left panel shows frequencies of cells with different numbers of detected barcodes. The dashed line shows the average number of detected barcodes per cell (2.85). The middle panel shows numbers of barcodes in relation to fluorescent reporter detection. Each dot represents one cell. The right panel shows frequencies of barcodes detected in different numbers of cells. The dashed line indicates that on average one barcode is detected in 1.54 cells. (**e**) Scar detection in iPSCs treated with no doxycycline, 2 µg of doxycycline for one day, and 2 µg of doxycycline for two days. (**f**) Bar plots showing percentage of scarred GFP transcripts detected in 3D cultures treated with 0-8 µg of doxycycline at EB and neuroectoderm stages (mean values +/− SEM). Dots show the values of individual samples.

We established a dual-channel lineage recorder called iTracer by coupling a complex barcode library together with an inducible Cas9 scarring system in iPSCs (Fig. 1a). The lineage recorder is based on the Sleeping Beauty transposon system, which enables efficient transposition^28^ of exogenous DNA into multiple genomic loci within iPSCs. A poly-adenylated green or red fluorescent protein (GFP or RFP) reporter is driven by the RPBSA promoter and contains a barcode (11 nucleotides of random bases) in the 3′ untranslated region, which serves as the first channel of the recorder. In the opposite direction, 91 bases away from the RPBSA promoter, we have introduced a human U6 promoter driving a guide RNA (gRNA) that targets a region in the 3′ portion of the sequence coding for GFP or RFP. This iTracer construct is introduced by electroporation into iPSCs that contain a doxycycline-inducible Cas9 cassette (iCRISPR)^29,30^. Fluorescent cells containing the reporter can be isolated using flow cytometry before being propagated or cryopreserved for later use (Extended Data Fig. 1a,b). From these uniquely barcoded iPSCs, we initiate cerebral organoids using approximately 2,000 cells to record lineages during organoid development (Fig. 1b). At a time point of choice, doxycycline introduction into the medium induces Cas9 expression in organoid cells, followed by formation of Cas9–gRNA complexes, leading to double-stranded break formation at the targeted location in the fluorescent reporter region of the recorder. These breaks are repaired by cellular machinery, resulting in insertions and/or deletions at the cut site, so-called scars, which serve as the second channel of our lineage recorder and can be read by sequencing the reporter transcript. Multiple barcodes and induced scars per cell could in principle be detected due to multiple insertions of the transposon-based reporter.

We measured barcode diversity using scRNA-seq data from three iTracer iPSC pools and found approximately 60,000 unique sequences (Methods). We followed barcode diversity throughout organoid development using targeted amplicon sequencing of the barcode region (Extended Data Fig. 1b,c) and found relatively stable diversity over the course of organoid development from pluripotency (Extended Data Fig. 1c). Further scRNA-seq characterization of an iTracer iPSC pool revealed 2.85 barcodes per cell; the average group size of the cells that shared the same barcode was 1.54 cells. Cells with a higher number of barcodes also had higher expression of iTracer fluorescent reporter mRNA (Extended Data Fig. 1d). We next analyzed the efficiency of Cas9-induced scarring at the embryoid body (EB) and neuroectoderm stages of cerebral organoid development by testing the duration and concentration of doxycycline treatment (Extended Data Fig. 1e,f). We found scarring was most efficient when samples were incubated with 8 µg ml^−1^ doxycycline for 24 h and applied these conditions in all subsequent experiments. Together, these data established suitable conditions for lineage-coupled single-cell transcriptomics in iPSC-derived cells and tissues using the iTracer system.

### iTracer reveals clonality of distinct cell populations

**Extended Data Fig. 2:**
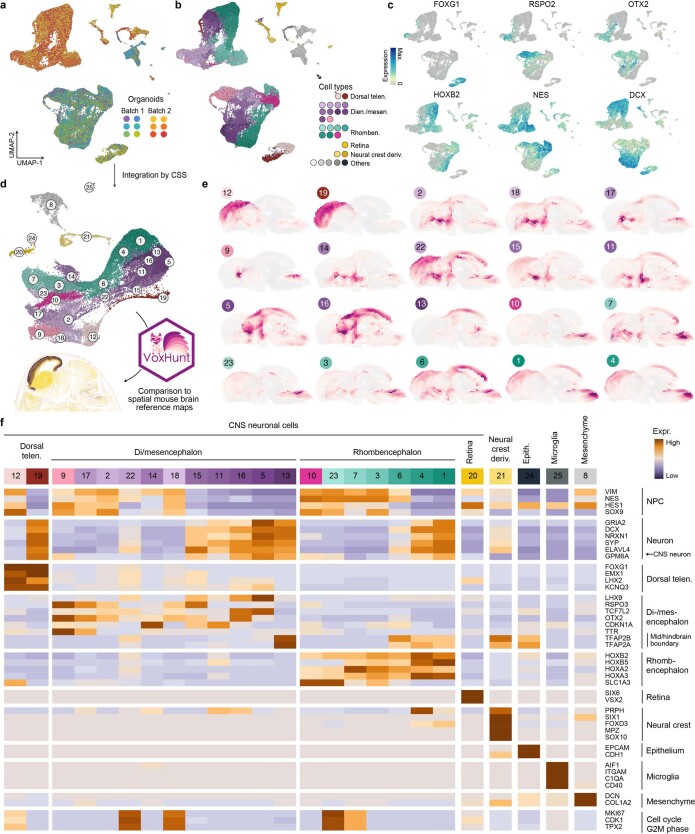
Integration and cell type annotation of iTracer cerebral organoid single-cell transcriptomes. (**a**) UMAP embedding of single-cells from two batches of iTracer whole-organoids without integration, colored by organoid. (**b**) UMAP embedding of single-cells from two batches of iTracer whole-organoids colored by cell type annotation (telen. - telencephalon; dien./mesen. - diencephalon/mesencephalon; rhomben. - rhombencephalon; neural crest deriv. - neural crest derivatives). (**c**) UMAP colored by expression of selected marker genes. (**d**) Schematic of cluster annotation of CSS integrated whole-organoid data using VoxHunt. Similarity scores are calculated between average gene expressions of identified clusters in the whole-organoid data and in situ hybridization signals in the E13.5 mouse brain in Allen Brain Atlas. (**e**) Sagittal projections colored by scaled similarity scores of each cluster from integrated whole-organoid data to voxel maps of the E13.5 mouse brain. (**f**) Heatmap of cell type and cell state marker genes across all CSS integrated whole-organoid clusters (mesen. - mesenchyme).

We next set out on a series of experiments using iTracer to study lineage dynamics during cerebral organoid development from pluripotency. Organoids were generated from approximately 2,000 iPSCs containing the iTracer recorder. We induced scarring at different organoid developmental time points (day 4, 7, 15, 20 or 30) and subsequently performed scRNA-seq after 1, 1.5 or 2 months of culture (Fig. 1b). Time points of scarring induction were selected around critical steps of organoid development including EB formation (day 4), neural induction (day 7), neuroepithelium development (day 15) and neurogenesis (days 20 and 30). We used cluster similarity spectrum (CSS) analysis^31^ to integrate 44,275 cells from 12 organoids across two batches (Fig. 1c, Extended Data Fig. 2a,b and Supplementary Tables 1 and 2), resulting in 25 cell clusters. Based on marker gene analysis and comparisons with primary reference atlases (Methods), 90% of cells were annotated to central nervous system cell types from the dorsal telencephalon, diencephalon, mesencephalon, rhombencephalon and retina, while a small fraction was annotated as non-brain populations including neural crest derivatives and mesenchymal cells (Fig. 1c,d and Extended Data Fig. 2c–f). Although proportions of cells with different regional identities varied among individual organoids, we detected all major cell populations in multiple organoids, consistent with previous reports using cerebral organoids^5,32^.

**Extended Data Fig. 3:**
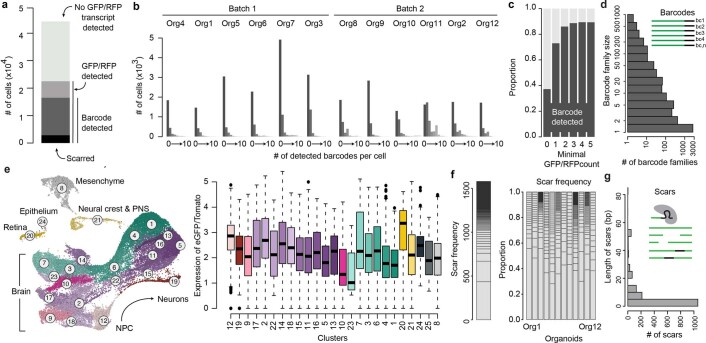
iTracer readouts across 12 organoids. (**a**) Stacked barplot showing the number of cells measured across all 12 organoids (lightest grey), where only iTracer reporter was captured (light grey), with reporter and barcodes (dark grey) or reporter, barcodes and scars (black). (**b**) Histograms of the cell numbers with different numbers of barcodes detected across all 12 organoids. (**c**) Stacked barplot showing the proportion of cells with barcode detected under different iTracer reporter transcript cutoffs. (**d**) Barplot of the number of barcode families and sizes of families detected. (**e**) UMAP embedding of scRNA-seq data of 44,275 cells from 12 cerebral organoids, cells are colored by cluster and boxplot of iTracer reporter expression (eGFP or Tomato) for each cluster in the corresponding UMAP projection. (**f**) Stacked bar plots showing frequency of unique scars among all 12 organoids. (**g**) Barplot of number of scars and scar lengths detected.

Overall, we detected iTracer readouts in 22,489 cells (51%) from this dataset (Extended Data Fig. 3a). Between organoids, we detected a variable number of reporter-expressing cells, barcodes and scars (Extended Data Fig. 3b and Supplementary Table 1). We noted that, while the entire starting population of iTracer iPSCs contained the reporter, transgene silencing and sparsity of scRNA-seq data are likely contributors to loss of reporter detection^33,34^. Nonetheless, of those cells in which the reporter transcript was detected by scRNA-seq, we identified at least one unique molecular identifier (UMI) covering the barcode region in 73% of cells (Fig. 1e and Extended Data Fig. 3c,d). Importantly, we found no significant associations of cell type or regional identity with lack of iTracer expression (Extended Data Fig. 3e). Due to the nature of the Sleeping Beauty system, cells can have multiple iTracer insertions, allowing for unique barcode and scar compositions in individual cells. In those cells in which at least one barcode was detected, we found an average of 2.10 barcodes per cell. Groups of cells that share the same barcode composition, termed barcode families, ranged in size from two to 801 and averaged approximately 11 family members. On average, 77.9% (batch 1) and 97.6% (batch 2) of barcode families were unique to a given organoid within a batch, indicating a limited overlap of highly diverse barcodes. CRISPR–Cas9 scars resulted in a similar proportion of insertion and deletion scar types, which varied in overall length (Fig. 1f and Extended Data Fig. 3f,g). Scarring efficiency varied per organoid (Supplementary Table 1); yet, of those cells in which we detected a barcode, 17% were also scarred. We found 237 groups of cells sharing the same scar and barcode composition, which were termed scar families.

**Extended Data Fig. 4:**
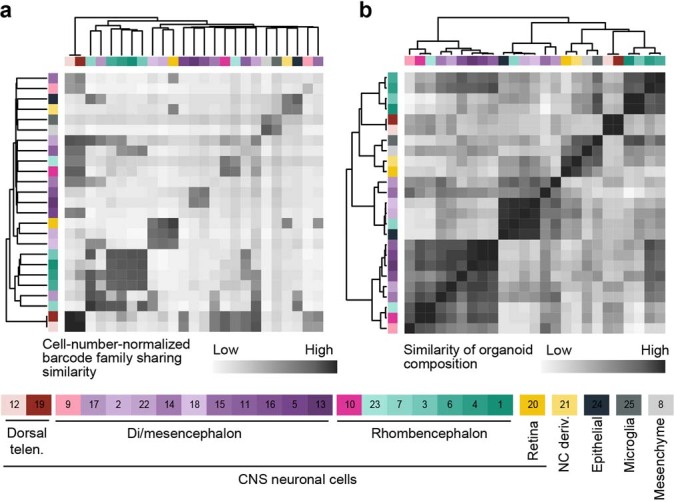
iTracer barcode families accumulate in distinct brain regions. (**a**) Heatmap of cell number normalized barcode family similarity, with hierarchical clustering (ward.D2 method) applied to cell-number-normalized barcode family composition distances. (**b**) Heatmap of similarity of organoid composition, defined as the Pearson’s correlation coefficient between cell frequencies of each cluster across different organoids, with hierarchical clustering (ward.D2 method) applied to the correlation distances between cell frequencies of clusters across different organoids.

We first focused on barcodes, which mark clones derived from one given cell in the initializing EB, and explored the clonality of transcriptionally distinct organoid cell clusters. We performed a permutation enrichment analysis using single cells from all lineage-traced organoids together to determine the likelihood that barcode families were shared between cell clusters (Fig. 1g and Extended Data Fig. 4a,b). We identified three groups of cell clusters with significant enrichment of distinct barcode clones. Each group labeled distinct brain regional identities, indicating that there was a robust trend during organoid development during which initiating iPSC clones accumulate in distinct brain regions. Importantly, these results could not be solely explained by differences in cell type composition between organoids. We illustrated regional barcode accumulation by projecting four different barcode families on the overall uniform manifold approximation and projection (UMAP) embedding and observed that iPSC clones were distributed into distinct brain regions or cell types (Fig. 1h,i).

### Spatial iTracer identifies clone enrichment in organoid regions

**Fig. 2:**
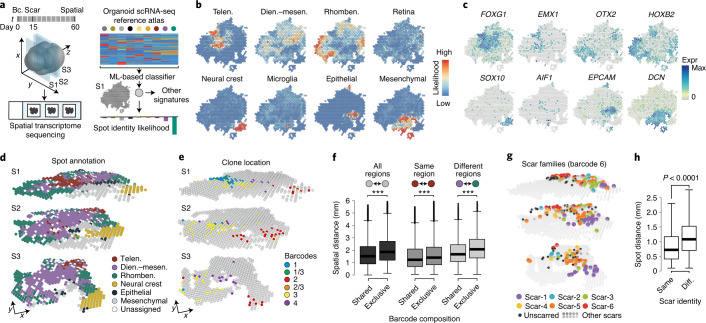
Spatial iTracer links lineage, molecular state and location information in cerebral organoids. **a**, Schematic of tissue section selection for Spatial iTracer. ML, machine learning. **b**,**c**, Spot plots for section 1 (S1) colored by projection likelihood to organoid reference clusters (**b**) or by marker gene expression (expr) (**c**). Dien.–mesen., diencephalon–mesencephalon. **d**,**e**, Spot plots for each section (S1–S3), colored by machine learning-based deconvolution for cell type assignment (**d**), or four example iTracer barcodes highlighting restriction of lineages to specific regions in the organoid (**e**). **f**, Box plots of pairwise spatial distance between spots found in the three sections, grouped by whether a shared barcode was identified by the spot pair. Regions that are spatially close to each other share similar barcode composition, whereas spots that are distant in space have decreased barcode similarity. This occurs when comparing any two spots, regardless of spot-annotated brain region (left), between any two spots in the same annotated brain region (middle) and between any two spots that are assigned to different annotated brain regions (right). Two-sided Wilcoxon rank-sum tests were performed comparing ‘shared’ with ‘same’ and ‘exclusive’ groups (*n*_shared_ = 53,618 (left), 14,698 (middle) and 18,200 (right) spot pairs from three slices; *n*_exclusive_ = 197,444 (left), 42,178 (middle) and 74,366 (right) spot pairs); ***unadjusted *P* value < 0.0001. **g**, Spot plot for each section colored by scar families within a single barcode. **h**, Box plots show the distributions of spatial distances between spots with the same scar or with different (diff.) scars. *P* values represent significance values from two-sided Wilcoxon tests (*n*_shared_ = 4,200, *n*_different_ = 24,268 spot pairs from three slices). Boxes in box plots represent upper and lower quartiles. The center line represents the median. Whiskers show the minimum and maximum of the data if there is no outlier. Outliers are defined as data points outside 1.5 times the interquartile range above the upper quartile and below the lower quartile.

**Extended Data Fig. 5:**
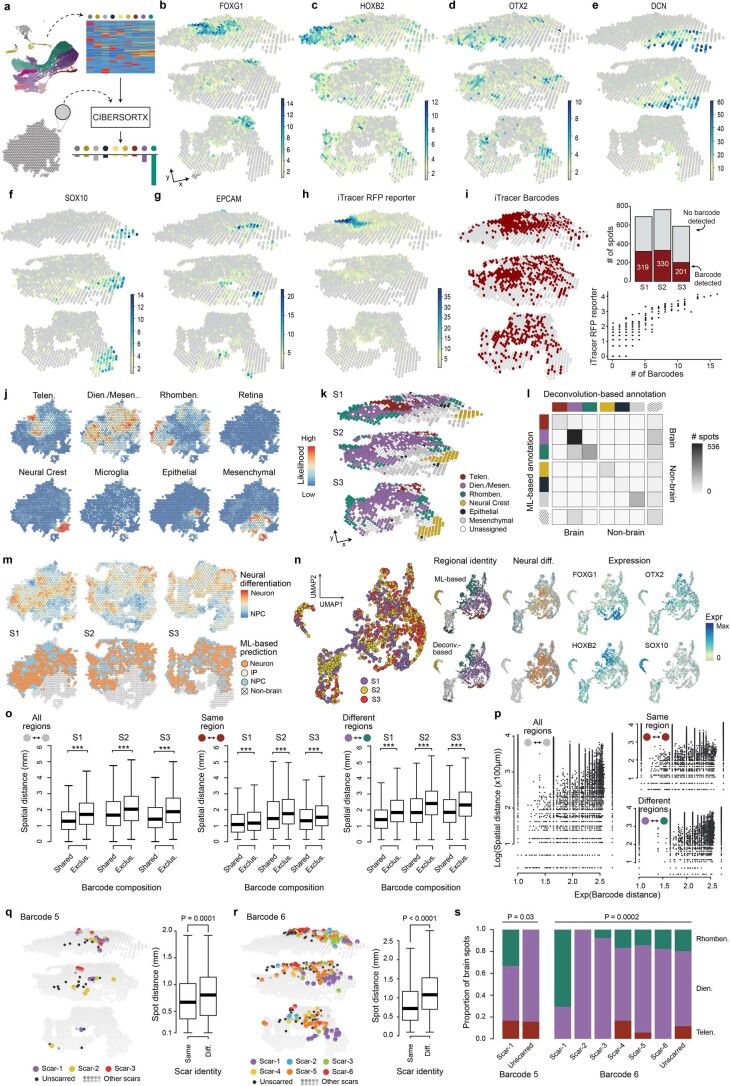
Regionalization of cell types across cerebral organoids. (**a**) Schematic of spot annotation by digital cytometry using CIBERSORTx. (**b**-**g**) 3D spatial feature plots of expression of genes marking different cell types and brain regions. (**h**) 3D spatial feature plots of expression of iTracer RFP reporter. **(i**) 3D spatial plot of spots with (red) and without (grey) iTracer barcodes detected. Barplot of detected iTracer barcodes across all sections. Scatterplot of detected barcodes and iTracer RFP reporter. (**j**) Spot plot for section 1 colored by deconvolved cell type assignment by CIBERSORTx. (**k**) Spot plot for each section (S1-S3), colored by deconvolved cell type assignment by CIBERSORTx (**l**) spot comparison between ML-based (Fig. 2b-d) and CIBERSORTx spot annotations (**m**) NPC and neuron scores plotted for section 1 (**n**) UMAP embedding of spots from sections 1-3 colored by regional identity, NPC/neuron scores, and expression of selected genes. (**o**) Boxplots of the iTracer barcode composition of each spot pair vs the spatial distance of each spot pair across all sections (S1-S3) in any regions (left), same cell type regions (middle) and different cell type regions (right). Two-sided Wilcoxon rank sum tests were performed comparing shared to same and exclusive groups, *** indicates p-values<0.0001 (n > 2898 spot pairs). (**p**) Scatter plots of barcode distance between each spot pair at the same section vs their spatial distance, in all the three sections, in any regions (left), same cell type regions (top-right) and different cell type regions (bottom-right). (q-r) 3D plot of spots across the three tissue sections where different scars on two example barcodes: barcode 5 (**q**) and barcode 6 (**r**) are highlighted. Boxplots show the distributions of spatial distances between spots with the same scar or with different scars. P values represent unadjusted two-sided Wilcoxon test significance (n_Shared_ = 298 (c), 4200 (d); n_Diff_ = 1206 (c), 24268 (d) spot pairs from three slices). (**s**) Stacked bar plots showing the proportion of spots with different annotated brain regional identities, with each bar representing spots with one barcode and one scar detected. P values show the χ^2^ test significance without adjustment.

We hypothesized that enrichment of barcodes in molecularly distinct cell populations with different brain regional identities may be associated with the spatial arrangement of clones throughout the organoid. To link molecular state, cell lineage and location information, we established ‘Spatial iTracer’, which uses spatial transcriptome sequencing based on the 10x Visium platform to measure gene expression and iTracer readouts. We applied Spatial iTracer to three intact 10-µm tissue sections derived from one 2-month-old cerebral organoid scarred during the neuroepithelium stage (day 15) (Fig. 2a and Supplementary Tables 1 and 4). Briefly, each tissue section was adhered to a slide with 6.5 × 6.5-mm capture areas containing several thousand spots, where each spot harbors millions of capture oligonucleotides each with a unique spatial barcode. Tissue sections were permeabilized to facilitate mRNA capture, and captured sequences were amplified and sequenced. We obtained transcriptome, barcode and scar topology of 2,038 spots, for which each spot covers an area of approximately one to ten cells. We used two independent methods (machine learning-based classification and CIBERSORTx^35^) to assign each spot to cell clusters identified in cerebral organoids using scRNA-seq (Fig. 2a and Extended Data Fig. 5a). Spots were assigned to the telencephalon, diencephalon–mesencephalon and rhombencephalon as well as non-neuronal identities, and both deconvolution methods showed agreement in region identity assignment (Fig. 2b–d and Extended Data Fig. 5b–l), suggesting that annotations were robust to computational approaches. Spots with the same annotation clustered within the same slice and persisted throughout the depth of the organoid, revealing transcriptome regionality. Spots showed varying levels of neural progenitor cell (NPC) or neuron signatures, revealing areas in the tissue slice enriched for progenitors and differentiated cells, respectively (Extended Data Fig. 5m). Regional and differentiation heterogeneity largely accounted for overall spot heterogeneity (Extended Data Fig. 5n). In total, 41.7% of spots contained at least one barcode, consistent with our iTracer data (Extended Data Fig. 5i and Supplementary Table 1). Spots with the highest barcode detection colocalized with the highest expression of the iTracer RFP reporter (Extended Data Fig. 5h).

We explored the spatial distribution of iTracer lineages and found that cells belonging to the same barcode family accumulated in distinct regions in the organoid slice, whereas cells belonging to different barcode families were spatially segregated (Fig. 2e,f and Extended Data Fig. 5o,p). The same spatial distribution was observed on the level of scar families, in which spots containing the same barcode–scar combination were found closer together and were more likely to belong to the same brain regional identity than those with the same barcode but different scars (Fig. 2g,h and Extended Data Fig. 5q–s). These data suggest that, during brain organoid development, related cells tend to accumulate in the same area of the organoid, receive similar patterning signals and therefore on average become restricted to the same brain regional identity. Together, iTracer and Spatial iTracer revealed an enrichment of cell clones in distinct brain regions of the cerebral organoid that can be traced back to clonality within the initializing EB.

### Lineage tracing with long-term light-sheet microscopy

**Fig. 3:**
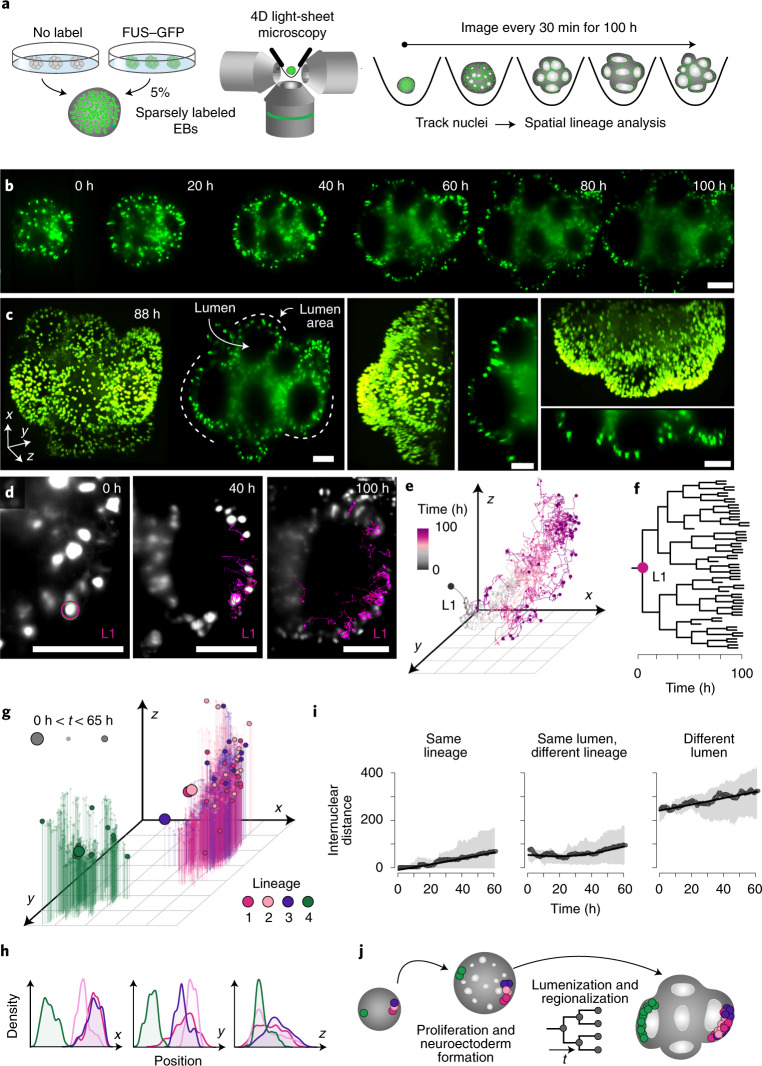
Long-term light-sheet microscopy of cerebral organoid development. **a**, Schematic showing the experimental setup. **b**, Cross-sectional (*x*–*y*) still images from time points at 0–100 h. Labeled nuclei are colored in green. **c**, Three-dimensional projection and cross-sections of *x*–*y*, *x*–*z* and *y*–*z* planes at 88 h. The empty lumen cavity and lumen areas are annotated on the image. **d**, Selected images show the increment in nuclei tracks of L1 from three time points over 100 h. Scale bar, 100 µm in all images (**b**–**d**). **e**, Three-dimensional plot showing spatial distribution of all nuclei in L1 over 100 h. **f**, Lineage tree across time for L1. **g**, Three-dimensional scatterplot of spatial distribution of nuclei from four lineages (L1–L4) traced over 65 h. Big, medium and small dots represent time points at 0 h and 65 h and times between 0 and 65 h, respectively. **h**, Density plot showing the distribution of all four lineages in *x*–*y*–*z* planes. **i**, Scatterplot showing internuclear distance between any two nuclei in the same lineage (L1, L2, L3 and L4), different lineages in the same lumen (L1–L3) and for nuclei in different lineages (L1–L3 and L4). Error bands show the first to 99th percentiles. **j**, Illustration shows proliferation and regionalization of tracked lineages in the organoid over 100 h.

**Extended Data Fig. 6:**
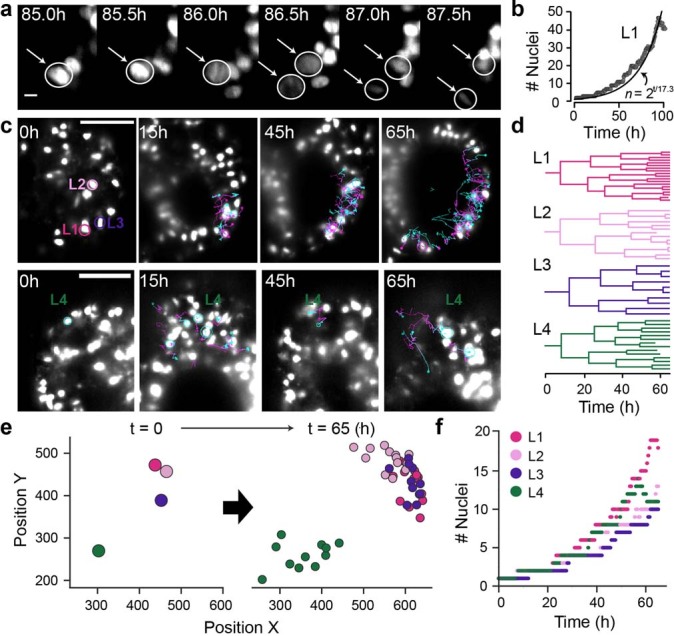
Tracking spatial distribution of nuclei lineages using Light sheet microscopy. (**a**) Example images show a nucleus dividing into two daughter nuclei in the developing organoid. White circles represent manual detection and tracking over time. (**b**) Scatter plot shows increase in the number of nuclei over 100 hours of tracking in lineage one (L1). The curve shows the exponential model estimated by the data, with the estimated doubling time being 17.3 hours. (**c**) Nuclei tracking of three lineages (L1-L3) in the same lumen area (top) and of a fourth lineage (L4) surrounding a diametrically opposite lumen (bottom). Scale bar is 100 µm in all images. (**d**) Lineage trees for the four tracked nuclei lineages. (**e**) Spatial distribution in the x-y plane of the four tracked lineages. Nuclei are shown at 0 hours and 65 hours, colored by lineage. (**f**) Dotplot shows the increase in the number of daughter nuclei for all four lineages over 65 hours.

To directly measure lineage dynamics and spatial accumulation of clones in a developing organoid during the neuroectoderm-to-neuroepithelium stage, we established long-term live imaging of developing cerebral organoids using 4D light-sheet microscopy (Fig. 3a, Extended Data Fig. 6a and Supplementary Videos 1 and 2). Briefly, we generated organoids containing 5% iPSCs that had nuclei labeled with a uniform fluorescent reporter, FUS–mEGFP^36^ and imaged the sparsely labeled organoids with an inverted light-sheet microscope (Fig. 3a). EBs were embedded in Matrigel in the imaging chamber and cultured in neural-induction medium, and development was tracked for 65–100 h (Fig. 3b and Supplementary Video 1). As the EB grew and developed, we observed the formation of several lumens, each of which could be tracked in three dimensions (Fig. 3c).

We directly tracked the lineage of a single nucleus throughout the recording time using Mastodon^37^, a framework allowing for semi-automated tracking and curation of nuclei lineages in large 4D datasets (Fig. 3d,e). We visualized the spatial distribution of daughter cells derived from the originating nucleus, which we call lineage 1 (L1), and generated a lineage tree resulting from 100 h of proliferation (Fig. 3f, Extended Data Fig. 6a and Supplementary Video 3). The average duration of one cell cycle was estimated to be 17.3 h (Extended Data Fig. 6b). We observed that L1 remained confined to the same area of the lumen throughout the recording time (Fig. 3d and Extended Data Fig. 6c). We tracked three additional nuclei, for which two nuclei were neighbors in the same lumen area as L1 (L2–L3) and the third nucleus (L4) was positioned diametrically opposite in a distinct future lumen area in the EB (Fig. 3g, Extended Data Fig. 6c,d and Supplementary Video 4). We quantified spatial distances between each tree and inspected the distribution of all daughter cells within the organoid 3D space (Fig. 3g–i). During the course of 65 h, the initializing nuclei gave rise to 13 descendant nuclei on average, which all populated the expanding organoid but remained spatially restricted to the parent lumen, exhibiting limited migration away from their lineage members (Fig. 3g–i and Extended Data Fig. 6e,f). These results suggest that early spatial arrangement of clones followed by local amplification results in distinct lineage compositions of brain regions, which confirms our previous iTracer-based observation of organoid brain region clonality (Fig. 3j).

### Lineage dynamics during cerebral organoid patterning

**Fig. 4:**
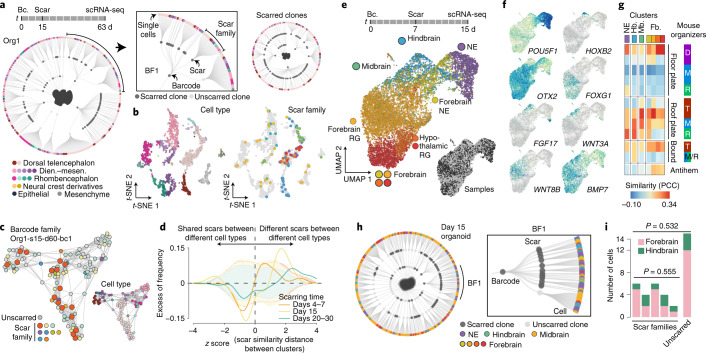
iTracer temporal lineage recording illuminates a window of fate restriction in cerebral organoids associated with brain patterning. **a**, Lineage plot shows full lineage reconstructions from a single organoid scarred at day 15 and sequenced at day 63 (left) as well as the subset of cells in which scars were detected (right). First- and second-order deviation nodes represent barcode and scar families, respectively, with terminal branches indicating individual cells. Each cell is colored based on the cell type designation. **b**, *t*-distributed stochastic neighbor embeddings (*t*-SNE) show cells for this organoid colored by both cell type annotation and scar families (≥5 members). **c**, Force-directed graph embedding of cells from a single barcode family within organoid 1 (Org1), with cells colored by scar family or cell type (inset). Scar families in orange show enrichment in cortical brain regions. **d**, Frequency distribution of *z* scores of scarring pattern distances between cell clusters after subtraction of background distribution estimated by random sampling of scars. Different scarring times are shown separately. Dashed shadow backgrounds show 90% confidence intervals, accordingly colored by scarring time. This plot highlights that scarring at later time points separates lineages with different cell fates. **e**, UMAP embedding of developing cerebral organoids scarred at day 7 and sequenced at day 15, with cells colored by cell types and estimated brain regional identity or samples (inset). RG, radial glia; NE, neuroepithelial. **f**, Expression of regional marker genes and selected morphogen genes. **g**, Heatmap of relative similarity of organoid clusters to organizing centers identified in developing mouse brains. T, telencephalon; D, diencephalon; M, mesencephalon; R, rhombencephalon; Hb., hindbrain; Mb., midbrain; Fb., forebrain; PCC, Pearson’s correlation coefficient. **h**, Lineage plot shows full lineage reconstructions from one day 15 organoid (sample 1). The zoomed-in view highlights a barcode family with diverse patterning states. **i**, Bar plot showing region composition of scar families, revealing that cells are not yet restricted at the time point of scarring at day 7. *P* values show results of two-sided Fisher’s exact tests to compare regional proportions in different scar families, with or without consideration of unscarred cells.

**Extended Data Fig. 7:**
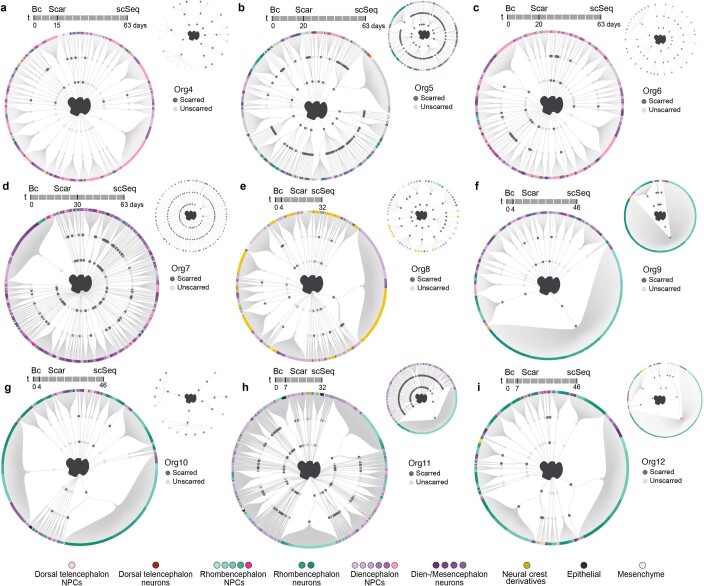
iTracer readouts enable construction of cell lineage trees from cerebral organoids. (**a**-**i**) Lineage plots show full lineage reconstructions, as well as the subset of cells where scars were detected from 9 organoids. The first and second order deviation nodes represent barcode and scar families respectively, with the terminal branches indicating individual cells. Each cell is colored based on the cell type designation.

Next, we used iTracer to determine when cells restrict their fate during brain organoid development. We used both channels of the lineage recorder (barcodes introduced in the initializing EB and scars induced during a developmental time course) together with single-cell transcriptomes to construct fate-mapped whole-organoid phylogenies (Fig. 4a,b and Extended Data Fig. 7a–i). We analyzed cell type diversity in scar families in which organoids were treated with doxycycline over a scarring time course. In an example of scarring induction at the neuroepithelial stage (day 15), we found one scar family restricted to a cortical fate, while other scar families were found distributed across different cell types (Fig. 4c). We globally analyzed scar families and found that fate restriction increased over the scarring time course, starting at day 15 (Fig. 4d). This analysis revealed a coarse patterning window for brain regionalization; however, sparse sampling of cells in whole organoids limits the depth of these analyses.

**Extended Data Fig. 8:**
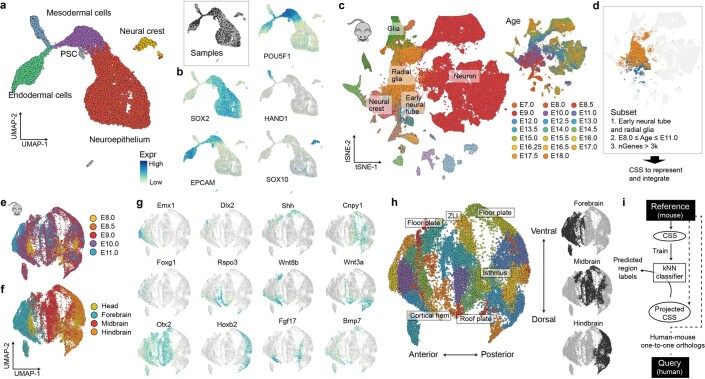
Early patterning in cerebral organoids. (**a**) UMAP embedding of all cells in the early cerebral organoids at day 15, with cells colored by annotated cell types or samples. (**b**) Expression of genes marking different cell types. (**c**) Overview of the developing mouse brain scRNA-seq atlas [La Manno preprint]. Cells are colored by annotated cell classes or sample ages. (**d**) A subset of cells, representing the early neural tube cells (ENTCs) and radial glia (RG) in different regions of the developing mouse brain, were selected and integrated using CSS. (**e**-**f**) UMAP embedding of mouse ENTCs and RG with cells colored by (e) sample ages and (f) dissected tissues. (g) Expression of genes marking different brain regions and genes encoding selected morphogens. (**h**) UMAP embedding of mouse ENTCs and RG with cells colored by cell clusters (left) or cluster regional identities (right). Labels show the organizer cells. (**i**) Schematic showing the computational workflow of projecting human early cerebral organoid cells to the developing mouse brain ENTCs and RG references to estimate their regional identities.

Guided by the observation of a patterning window, we investigated gene expression heterogeneity at the neuroepithelial stage (day 15) using iTracer organoids scarred at the neuroectoderm stage (day 7) (Fig. 4e and Extended Data Fig. 8a,b). To interpret expression patterns at this early time point, organoid cells were projected to an atlas of radial glia in the primary prenatal mouse brain^38^ (Extended Data Fig. 8c–h) based on a k-nearest-neighbor classifier trained on the mouse atlas CSS representation^31^ (Extended Data Fig. 8i). The projection revealed regionality in the neuroepithelium at day 15 and strong correspondence between human and mouse patterning profiles (Fig. 4f,g). Consistent with our previous observation of regional clonality, barcode families showed distinct brain region compositions (Fig. 4h). However, in the largest barcode family containing the highest regional diversity (BF1), we found no significant difference in regional identity composition among different scar families (Fig. 4i). Our results imply that brain regional heterogeneity starts to emerge after the neuroectoderm stage and before the neuroepithelium stage. Altogether, these data provide molecular profiles of nascently regionalized human neuroepithelium and lineage information at the onset of brain patterning in human cerebral organoids.

### Variability of lineage dynamics in individual organoid regions

**Fig. 5:**
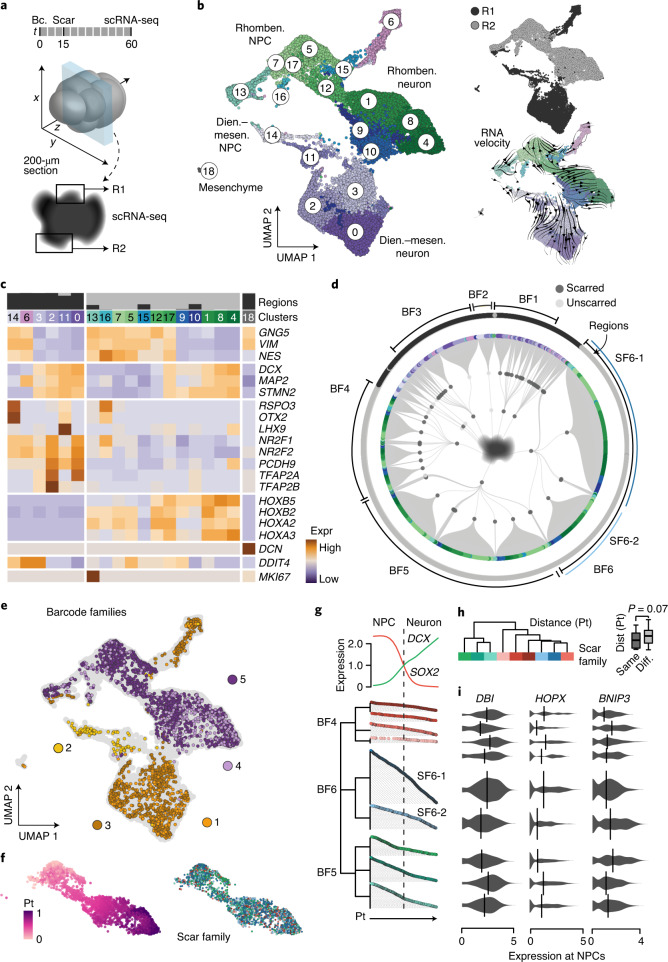
iTracer identifies distinct neurogenic lineage families in individual cerebral organoid regions. **a**, Schematic of tissue section selection for deep sampling. One 200-µm section was cut before selecting two spatially distant regions for microdissection. Single cells were isolated from microdissected regions and processed for scRNA-seq separately. **b**, UMAP embedding of scRNA-seq data from 26,894 cells from two microdissected regions in a single cerebral organoid scarred at day 15 and sequenced at day 60; cells are colored by cluster or originating region and annotated with brain regional or cell type identity. **c**, Expression heatmap showing brain region and cell type markers across clusters and regions shown in **b**. **d**, Lineage plot shows lineage reconstruction combining both microdissected regions from the single organoid. First- and second-order deviation nodes represent barcode and scar families (SF), respectively, with terminal branches indicating individual cells. The originating region is annotated in the outer circle, with example barcode and scar families annotated. **e**, UMAP embedding of single cells from the two microdissected regions colored by example barcode families indicated in **d**. **f**, Pseudotime (Pt) analysis was applied to cells from dissected region R2 (left) and colored by the nine scar families with at least 50 cells (right). **g**, Pseudotime distribution of cells in different scar families. *SOX2* (red) and *DCX* (green) expression patterns along the reconstructed pseudotime are shown (top). The dendrogram shows lineage reconstruction from barcode and scar families. Cells in each scar family are ordered by pseudotimes. **h**, Hierarchical clustering of scar families based on their pairwise distances (dist) on the pseudotime distribution. The box plot shows the distribution of distances between scar families from the same barcode family (left) or different barcode families (right). The *P* value indicates the significance score from two-sided Wilcoxon’s rank-sum test (*n*_1_ = 29, *n*_2_ = 81 scar families). Boxes in box plots represent upper and lower quartiles. The center line represents the median. Whiskers show the minimum and maximum of the data if there is no outlier. Outliers are defined as data points outside 1.5 times the interquartile range above the upper quartile and below the lower quartile. **i**, Examples of genes with differential expression among NPCs from different scar families.

**Extended Data Fig. 9:**
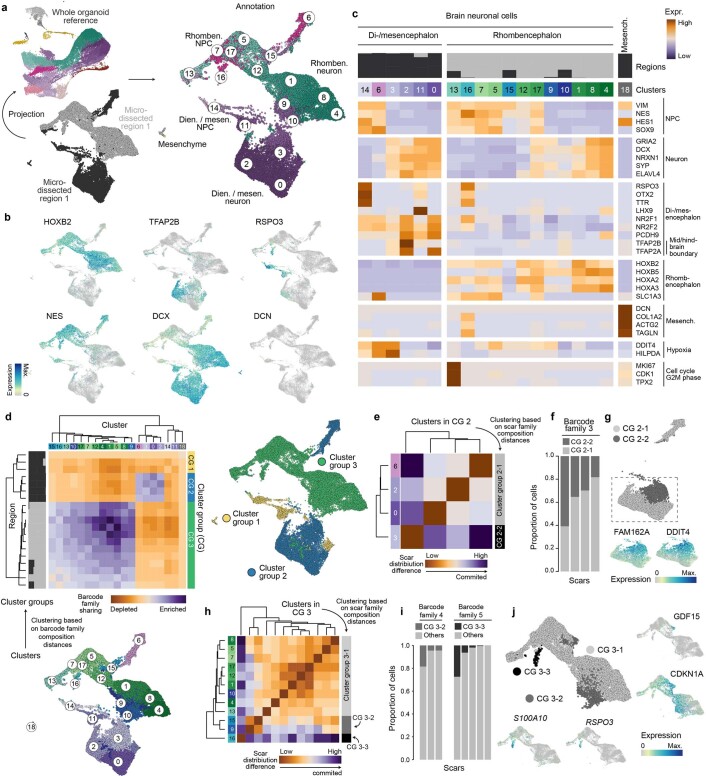
Deep cell and lineage sampling of micro-dissected regions from cerebral organoids. (**a**) Schematic of data projection procedure for single-cells from two regions of a micro-dissected organoid to the whole-organoid scRNA-seq data to assist cell annotation. UMAP embedding of single cells colored by projected annotation from the whole-organoid scRNA-seq data. (**b**) Expression of selected cell type markers in cells in the two regions of the micro-dissected organoid. (**c**) Heatmap of cell type and cell state marker genes across all micro-dissected organoid clusters. (**d**) Hierarchical clustering of the micro-dissected organoid clusters, based on the barcode family composition distances. UMAP embedding of single-cells is colored by the resulting three groups of clusters (CG.1-CG.3). Clusters in each group share similar barcode family compositions. (**e**) Hierarchical clustering of cluster group 2 (CG.2) based on scar family composition distance, to identify two subgroups of clusters. Clusters in each of the subgroups share similar scar family compositions. (**f**) Stacked bar plots showing distributions of cell proportions across subgroups of CG.2 clusters with distinct scar family compositions. Each stacked bar shows a different scar family in the same example barcode family 3. (**g**) UMAP embedding of cells in CG.2 clusters, colored by the two cluster subgroups with distinct scar family compositions, and expression of example genes with differential expression between the two subgroups. (**h**) Hierarchical clustering of cluster group 3 (CG.3) based on scar family composition distance, to identify three subgroups of clusters. Clusters in each of the subgroups share similar scar family compositions. (**i**) Stacked bar plots showing distributions of cell proportions across subgroups of CG.3 clusters with distinct scar family compositions. Each stacked bar shows a different scar family in the same example barcode family 4 (left) or barcode family 5 (right). (**j**) UMAP embedding of cells in CG.3 clusters, colored by the three cluster subgroups with distinct scar family compositions, and expression of example genes with differential expression between the two subgroups.

Next, we wished to use iTracer to assess variability in progenitor-to-neuron lineages in distinct brain organoid regions at high resolution. To achieve deep lineage sampling, we performed lineage-coupled single-cell transcriptomics on two microdissected peripheral regions of a 200-µm iTracer organoid section (2 months, scarred at day 15) (Fig. 5a and Supplementary Tables 1 and 3). Analysis of single-cell transcriptomes revealed region 1 (R1) as the diencephalon–mesencephalon and R2 as the rhombencephalon (Fig. 5b,c and Extended Data Fig. 9a–c). We reconstructed barcode and scar families and found that lineages in R1 and R2 were entirely diverged (Fig. 5d,e). We grouped transcriptionally distinct clusters based on barcode families (Extended Data Fig. 9d) and applied hierarchical clustering within these cluster groups (CGs) based on their scar family composition. This analysis revealed that CGs contain distinct scar family compositions and transcriptomic signatures (CG2-1, CG2-2, CG3-1–CG3-3; Extended Data Fig. 9e–j), indicating cell fate restriction before neuroepithelium formation at day 15. Notably, trajectory inference and RNA velocity analysis were not able to resolve distinct lineage relationships that could be distinguished using iTracer (for example, cluster 6, BF3; Fig. 5b and Extended Data Fig. 9g)

To compare neurogenic lineages within the same brain region, we ordered cells of all hindbrain scar families (R2) along a differentiation pseudotime using the transcriptome portion of the data (Fig. 5f,g). We found that sister lineages, for example, scar families belonging to the same barcode family and hence derived from the same barcoded cell in the EB, showed similar NPC-to-neuron distributions along pseudotime, indicating synchrony in differentiation dynamics (Fig. 5h). By contrast, non-sister lineages showed larger variability in the distribution of cells along pseudotime and hence less synchronized differentiation of NPCs to neurons. We next compared neurogenic lineages on a transcriptional level and identified a limited set of genes differentially expressed between different lineages (Fig. 5i). However, sister lineages did not show greater transcriptional similarity than non-sister lineages (two-sided Wilcoxon’s rank-sum test, *P* = 0.25).

### Tracing perturbed lineages with iTracer-perturb

**Fig. 6:**
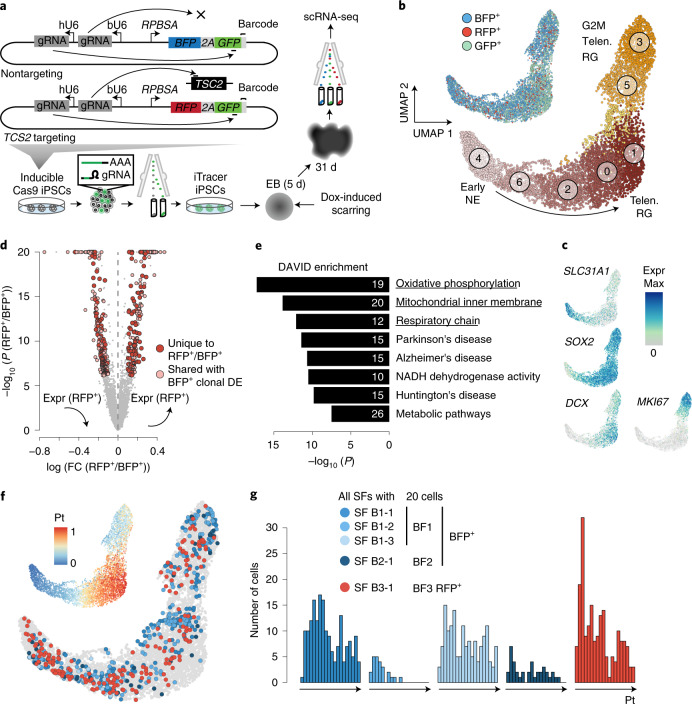
iTracer-perturb allows for simultaneous genetic perturbation and lineage tracing in mosaic organoids. **a**, Schematic for *TSC2*-targeting or control iTracer-perturb vectors. This system was applied to *TSC2* in mosaic cerebral organoids. **b**, UMAP embedding of scRNA-seq data from 1,673 RFP^+^, 4,766 BFP^+^ and 7,992 GFP^+^ cells sorted from one mosaic iTracer-perturb organoid at 1 month. Cells are colored by cell clusters (bottom) or fluorescent signals (top). **c**, Feature plot shows marker gene expression. **d**, Volcano plot shows overall differential expression analysis between RFP^+^ (*TSC2*-targeting) and BFP^+^ (control) cells, resulting in 385 DEGs (red), 197 of which are unique to the RFP^+^ versus BFP^+^ comparison (dark red). FC, fold change; DE, differentially expressed. **e**, DAVID functional enrichment analysis of DEGs with significantly increased expression that was uniquely observed in RFP^+^ cells. Bars show −log_10_ (*P*) values from tests, and numbers show the number of DEGs annotated with each term. **f**, UMAP with cells colored by their scar families (≥20 members) and fluorescent signals or by pseudotime (inset). **g**, Distribution of cells from different scar families (≥20 members) across the constructed pseudotime course.

The ability to record lineages and measure molecular states simultaneously in high throughput is particularly interesting when studying the effect of disease-associated genetic mutations on development. We therefore extended the iTracer system to enable simultaneous targeted gene perturbation, lineage recording and molecular state phenotyping in mosaic organoids. We introduced a second gRNA under the control of a bovine U6 promoter into the iTracer vector, which can target any gene of interest. In addition, we incorporated a second fluorescent reporter linked through a 2A self-cleaving peptide sequence to the barcoded GFP, which is not targeted by a scarring guide and therefore enables selection of lineage-recorded cells. iTracer-perturb targeting vectors contain gRNA species targeting the gene of interest (bU6) and *GFP* (hU6) as well as RFP and barcoded GFP reporters linked through the 2A peptide. iTracer-perturb nontargeting vectors contain a control dummy gRNA (bU6) and a gRNA targeting *GFP* (hU6) as well as *BFP* and barcoded GFP reporters linked through the 2A peptide (Fig. 6a). We designed an experiment in which cells within the same mosaic organoid would either be wild type or would carry targeting or nontargeting recording vectors. GFP scarring and target gene knockout could then be induced simultaneously at a time point of choice by doxycycline-mediated induction of Cas9 expression in the developing organoid.

**Extended Data Fig. 10:**
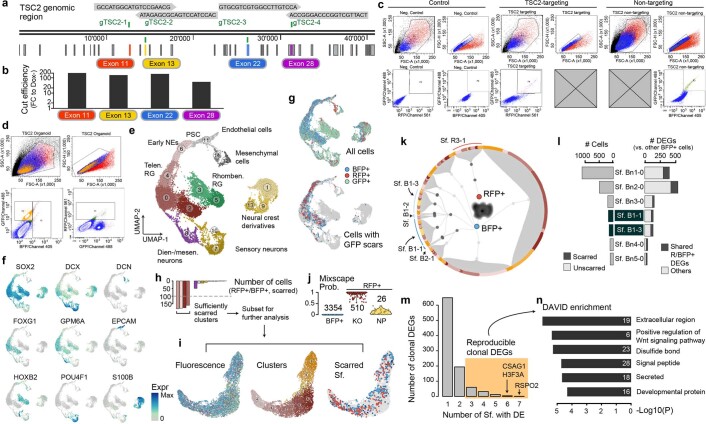
Simultaneous TSC2 perturbation and lineage tracing with iTracer. (**a**) Schematic of gRNAs designed to target the TSC2 gene. (**b**) Bar plots of cut efficiency, represented as fold changes of modified read proportions to negative controls, at the predicted target site when different gRNAs were lipofected. (**c**) FACs plots of sorting scheme used to isolate TSC2-targeting and non-targeting perturb-iTracer positive cells. Cells were gated first based on the main population to avoid debris, followed by gating for single cells excluding doublets, and lastly sorted for GFP + RFP positive (TSC2-targeting) or GFP + BFP positive (non-targeting) cells as compared to non-fluorescent negative controls. (**d**) FACs plots of sorting scheme used to isolate cells in the TSC2-perturb-iTracer organoid. Cells were firstly gated similar to (c), and lastly sorted for GFP-only positive, RFP positive and BFP positive cells. (**e**-**g**) UMAP embeddings show the complete scRNA-seq data in the cerebral organoid, with cells colored by (e) their cell clusters and annotations, (f) expression of selected marker genes, and (g) fluorescence of all cells or only cells with GFP scars. (**h**) Numbers of RFP + /BFP + cells with GFP scars in each cell cluster. The dash line shows 100 cells. Clusters with at least 100 RFP + /BFP + cells with GFP scars are considered as sufficiently scarred clusters. (**i**) UMAP embeddings show cells in the sufficiently scarred clusters, colored by their fluorescence, cell clusters and scar families (>=20 members). (**j**) Mixscape-estimated posterior probability of perturbation, with BFP + cells as non-targeting and RFP+ cells as targeting. The numbers show the cell number estimated in each category. (**k**) Lineage plot of cells in the sufficiently scarred clusters. The first order deviation nodes represent BFP+ and RFP+ cells; second and third order deviation nodes represent barcode and scar families respectively. The terminal branches indicate individual cells. Each cell is colored based on the cell cluster designation. (**l**) Numbers of cells in different BFP + scar families, and numbers of DEGs identified between cells in each BFP+ scar family and other BFP + cells. (**m**) Frequency of genes identified as DEGs between different BFP+ scar families. Genes detected as DEGs in at least three BFP+ scar families are defined as reproducible clonal DEGs. (n) DAVID functional enrichment of reproducible clonal DEGs.

We targeted the gene *TSC2*, which encodes a negative regulator of mTOR; somatic mutations in this gene have been shown to underlie certain focal cortical dysplasias in humans^25–27^. We created four targeting guide vectors, each containing a gRNA targeting a different exon in the *TSC2* gene (Extended Data Fig. 10a,b). We generated mosaic cerebral organoids composed of 10% iTracer-perturb control cells (BFP^+^GFP^+^), 10% iTracer-perturb *TSC2*-targeting cells (RFP^+^GFP^+^) and 80% wild-type cells (Extended Data Fig. 10c). We induced Cas9 expression during the EB stage (day 5), thereby creating *TSC2* perturbations and GFP scars simultaneously. At 1 month, we dissociated the mosaic organoids and used flow cytometry to sort reporter-positive cells, followed by scRNA-seq (Fig. 6a and Extended Data Fig. 10d–g). Focusing on scarred cell populations (Fig. 6b,c and Extended Data Fig. 10h,i), we performed differential expression analysis between cells with targeting (RFP^+^) and nontargeting (BFP^+^) vectors and between nontargeting cell clones as background (Fig. 6d and Extended Data Fig. 10j–l). We identified 197 differentially expressed genes (DEGs) that were unique to *TSC2*-targeting cells. Functional enrichment analysis of these genes using the database for annotation, visualization and integrated discovery (DAVID) revealed oxidative phosphorylation, the mitochondrial inner membrane and the respiratory chain as enriched categories (Fig. 6e, Extended Data Fig. 10m,n and Supplementary Table 8), consistent with previous reports of *TSC2* ablation affecting mitochondria via mTOR activation^39^. We combined lineage information with pseudotemporal ordering to compare the distribution of cells over pseudotime in different lineages (Fig. 6f,g). Importantly, *TSC2*-targeted lineages (RFP^+^) were enriched for early neuroepithelial cells (NECs) relative to control cells (BFP^+^) (odds ratio = 1.5; Fisher’s exact test, *P* = 0.03), suggesting a role of *TSC2* during early differentiation into NPCs. This is consistent with previous reports of delayed neuronal differentiation in patient-derived *TSC2*-heterozygous NPCs^40^. Together, incorporating gene perturbation into the iTracer lineage-recording system provides a flexible method to explore mosaic loss-of-function mutations in human organoids to understand mechanisms of developmental disorders.

## Discussion

Here we established three new approaches to trace lineages in human iPSC-derived organoids. First, we present iTracer, a dynamic dual-channel cell lineage recorder for human iPSC-derived organoids designed to trace clones from an initializing iPSC pool as well as to dynamically trace lineages using inducible scarring. Second, we present Spatial iTracer, which links molecular state, lineage and location information in organoids. Third, we accomplished direct lineage tracking using long-term light-sheet microscopy with mosaic fluorescent organoids. Importantly, all of these lineage-recording approaches, along with computational and data-visualization methods that we developed, can be applied to any human iPSC-derived cell differentiation or organoid system. We use these approaches to provide an extensive examination of lineage dynamics in developing cerebral organoids. We observed accumulation of cell lineages in distinct molecularly and spatially defined regions, which we could trace back to spatial restriction of lineages in the neuroepithelium before patterning. This brain regional clonality is consistent with fate maps in *Drosophila*^41^, zebrafish^42–44^ and mice^45^, which revealed that brain regionalization initiates early in development, and, as NECs do not migrate very far from where they are born, cells related to each other by lineage tend to contribute to the same part of the brain. Importantly, our findings corroborate results from the analysis of somatic mutation landscapes in different regions of the adult human brain, which also revealed clonal relationships linked to brain regions^46^. Cerebral organoids therefore represent a powerful system to systematically study the relationship between regionalization and clonal dynamics during human brain development.

Transgene silencing, single-cell dropout and limited cell sampling are hurdles in collecting complete lineage information using the iTracer system. Nonetheless, we were able to successfully reconstruct sparse lineages in whole organoids at various stages of development and observe a coarse timing for cell fate restriction. Microdissection enabled deeper lineage sampling, identifying different differentiation states that were derived from the same lineage for many different lineages. This strategy can be used to search for differences in gene expression between lineage families as well as differences in the timing of differentiation events. We have observed that indirect trajectory reconstructions from transcriptomes can give unreliable state flows, and methods that directly measure lineage are important to confirm trajectory inferences.

The iTracer-perturb system enables inducible or temporally controlled gene perturbations while also recording lineage. As a proof of principle, we targeted *TSC2* immediately before neuroectoderm formation, as mutations in this gene have been shown to underlie certain focal cortical dysplasias in humans^25–27^. Our results indicated that clones carrying the TSC2 gRNA have differential gene expression profiles associated with metabolic changes as compared to control counterparts, which is consistent with the role of TSC2 as negative regulator of mTOR, a master regulator of cellular metabolism. More generally, iTracer-perturb provides a strategy to simultaneously perturb, trace and measure states in any mosaic organoid system. In the future, iTracer and iTracer-perturb, together with long-term 4D light-sheet imaging, will be powerful methodological approaches to understand the effect of mutations that underlie developmental disorders in human organoid systems.

## Methods

### Ethics statement

Permission for this work with human iPSC lines was obtained through the Sachsisches Staatsministerium fur Umwelt und Landwirtschaft (Az. 55-8811.72/26, Az. 55-8811.72/26/382, Az. 55-8811.72/26/393 and 54-8452/26/7), the Swiss Federal Office for the Environment (A120821-08, A192559-01), the Ethics Committee of Northwest and Central Switzerland (2019-01016) and the Swiss Federal Office of Public Health.

### iCRISPR cell line

We used the iCRISPR iPSC cell line with doxycycline-inducible Cas9, created previously as described^29^. Briefly, the human iPSC line h409b2 from the RIKEN BRC cell bank^4^ was used to create a doxycycline-inducible Cas9-expressing cell line^30^ by introducing two transcription activator-like effector nucleases targeting the *AAVS1* locus, which has shown to be effective for sustained transgene expression, and two constructs encoding transcription activator-like effector nucleases with donor plasmids. One of the donor plasmids contained a constitutive reverse tetracycline transactivator (AAVS1-Neo-M2rtTA), and the other one contained a doxycycline-inducible expression cassette (Puro-Cas9). A mutation corresponding to D10A was introduced by site-directed mutagenesis of the original Puro-Cas9 donor with the Q5 mutagenesis kit (New England Biolabs, E0554S) to generate Cas9n. The cell line was tested for the proper expression of pluripotency markers SOX2, OCT-4, TRA1-60 and SSEA. Quantitative PCR confirmed the doxycycline-inducible Cas9n, and digital PCR was used to exclude off-target integration^29^. DNA from iCRISPR cells was sent to the Cell Guidance Systems Genetics Service Cytogenetics Laboratory and tested for copy number changes using the Agilent ISCA 8x60K version 2 array. Array analysis revealed three apparently clonal changes in DNA copy number. Gain of the entire long arm of chromosome 1 was detected and estimated to be present in about 70% of cells. A DNA copy number loss of approximately 3.7 Mb from the proximal short arm of chromosome 19, band p12, was detected and present in about 30% of cells. A commonly found mutational gain of approximately 1 Mb was detected within the proximal long arm of chromosome 20, band q11.21. Finally, applying inferCNV (https://github.com/broadinstitute/inferCNV) to the scRNA-seq data of iCRISPR suggested the gain of a large portion of chromosome 12 in a subset of cells. iCRISPR cells were regularly tested for mycoplasma using PCR validation (Venor GeM Classic, Minerva Biolabs) and found to be negative.

### Establishment of dynamic cell lineage-reporter vector

iTracer plasmids were constructed by modifying Sleeping Beauty reporter plasmids pSBbi-GH and pSBbi-RH^28^. pSBbi plasmids were a gift from E. Kowarz (Institute of Pharmaceutical Biology, Goethe University, Frankfurt am Main, Germany) (Addgene plasmids 60514 and 60516; http://n2t.net/addgene:60514, http://n2t.net/addgene:60516; RRID, Addgene_60514; RRID, Addgene_60516). Plasmid generation consisted of two steps: initially removing the hygromycin cassette and inserting an 11-bp barcode tag in the 3′ untranslated region of the fluorescent reporter gene; second, the gene-of-interest site including its promoter region was replaced by the human U6 promoter driving the expression of a gRNA that targets the fluorescence gene. In detail, a long-range PCR was performed to amplify and extract the plasmid backbone excluding the hygromycin cassette from pSBbi plasmids following the manufacturer’s recommendations (1× Phusion HiFi Ready Mix, 50 ng pSBbi, 0.2 µM of each primer (primer sequences are provided in Supplementary Table 5), 3% DMSO, 50 µl in total, 25 cycles). To remove original plasmid from the backbone, the PCR reaction was digested with a combination of restriction enzymes directly added to the PCR reaction (37 µl H_2_O; 10 µl Thermo Fisher FastDigest (FD) Buffer; 1 µl each FD enzyme DpnI, CpoI and Esp3I; 37 °C; 1 h). Reactions were purified using the Qiagen PCR Purification kit. The purified backbone was then used to perform Gibson assembly following the instructions in the CROP-seq manual^47^ to introduce barcodes by using an oligonucleotide containing random nucleotides (Supplementary Table 5). Instructions from the manual were only adapted such that bacteria were not plated after recovery but were instead completely transferred to an overnight culture with 1× ampicillin to maintain barcode heterogeneity. Plasmid DNA was isolated using the Qiagen Miniprep kit. CROP-seq plasmids containing a gRNA targeting either sequences for dTomato (GGTGTCCACGTAGTAGTAGC) or GFP (TGTTCTGCTGGTAGTGGT) were generated following protocol instructions (oligonucleotide sequences are provided in Supplementary Table 5). These plasmids acted as templates to amplify and extract the human U6 promoter, gRNA and the gRNA scaffold region using the PCR conditions described above. Primers (Supplementary Table 5) compatible with subsequent cloning were used, and the purified PCR product was digested with Thermo Fisher FD BshTI and FD PaeI before ligation. The backbone for cloning was obtained by digesting plasmids containing barcodes with Thermo Fisher FD BshTI and FD PaeI. To guarantee that there was no ligation of the cut region back to the plasmid, the backbone digest was run on an agarose gel, and the backbone region was excised and purified. Backbone and insert were ligated using NEB QuickLigase, and *Escherichia coli* was subsequently electroporated with the ligation product by following instructions in the CROP-seq manual. Recovered cells were again directly transferred to an overnight culture, and plasmid DNA was extracted and purified using Qiagen MediPrep.

### Assessment of scarring efficiency

To test scarring efficiency upon doxycycline induction, iCRISPR cells were cultivated using standard feeder-free conditions in mTeSR1 (Stemcell Technologies) on Matrigel-coated plates. Cells were nucleofected with 10 μg lineage-recorder DNA and 1 µg Sleeping Beauty transposase following the manufacturer’s protocol and using the B-16 program of the Nucleofector 2b (Lonza) in cuvettes for 100 µl Human Stem Cell nucleofection buffer (Lonza, VVPH-5022). Nucleofection reactions were plated on Matrigel-coated plates and allowed to expand for 5–7 d before flow cytometry. To select for cells with successful integration of the cell lineage reporter, RFP^+^ or GFP^+^ cells were sorted into 1.5-ml tubes (~120,000 cells in total) and plated in 12-cell Matrigel-coated plates with mTeSR1 and Rock inhibitor (1:250). Following cell recovery, we used 2 µg doxycycline^30^ for 0, 1, or 2 d before changing back to mTeSR1 base medium. Optimization of the incubation of 3D cultures with doxycycline was performed at EB and neuroectoderm stages at concentrations of 0.5, 1, 2, 4 and 8 µg ml^−1^ doxycycline for 24-h incubations before changing back to mTeSR1 and NIM base media, respectively. iPSCs were collected using Accutase (Sigma-Aldrich) for 5–7 min before quenching with KnockOut Media (Thermo Fisher Scientific) and centrifuging at 200*g* for 5 min. In total, 100,000 iPSCs were taken for DNA extraction using QuickExtract (Lucigen), whereas organoid samples were directly added to QuickExtract (Lucigen) and vortexed. All samples were vortexed shortly before heating to 65 °C for 6 min before vortexing again and heating to 98 °C for 2 min. We used 50 ng input DNA for scar region amplification (primer sequences are provided in Supplementary Table 5). Quality of the amplified product was checked with a 2% E-Gel (Thermo Fisher Scientific) before Illumina sequencing adaptors were added in a subsequent PCR reaction. Bulk scar libraries were cleaned with magnetic beads (Beckman Coulter) before checking quality with a 2% E-Gel. Libraries were sequenced on the Illumina MiSeq Nano. Scar detection was performed using CRISPResso^48^.

### Preparation of organoids and scarring

iTracer^+^ iCRISPR cells were prepared as previously described above. Following cell recovery after flow cytometry, 2,000 cells per well in a 96-well plate were seeded and differentiated into cerebral organoids using a whole-organoid differentiation protocol^5,22^. Throughout development, organoids were scarred by activation of inducible Cas9 (Fig. 1b and Supplementary Table 1). Scarring was achieved by first selecting the organoid to be scarred and transferring it to a six- to 24-well plate (depending on organoid size) filled with scarring medium with 8 µg ml^−1^ doxycycline (base medium depending on the age of the organoid, Supplementary Table 1). Organoids were incubated in scarring medium for 24 h before returning to base medium without doxycycline.

### Bulk barcode detection

IPSCs and 19 organoids ranging in stage from EB to day 30 were used for bulk analysis to assess the capture and diversity of iTracer barcodes. We propagated and collected samples in a manner similar to that described above. Briefly, iCRISPR cells were cultivated using standard feeder-free conditions in mTeSR1 (Stemcell Technologies) on Matrigel-coated plates. Cells were nucleofected with 10 µg lineage-recorder DNA and 1 µg Sleeping Beauty transposase following the manufacturer’s protocol and using the H9 program of the 4D-Nucleofector (Lonza) in cuvettes for 100 µl Human Stem Cell nucleofection buffer (Lonza, VVPH-5022). Nucleofection reactions were plated on Matrigel-coated plates and allowed to expand for 5–7 d before flow cytometry. To select for cells with successful integration of the cell lineage reporter, RFP^+^GFP^+^ cells were sorted into 1.5-ml tubes (~120,000 cells in total) and plated in 12-cell Matrigel-coated plates with mTeSR1, Rock inhibitor (1:250) and Primocin (1:250). DNA isolation and bulk barcode libraries were prepared as described for bulk scar libraries (primer sequences are provided in Supplementary Table 5). Libraries were sequenced on the Illumina MiSeq Nano. Analysis was performed using a custom Perl script to count the frequency of each uniquely detected barcode.

### Preparation of single-cell transcriptomes from whole lineage-traced organoids

Whole organoids were dissociated to generate single-cell gene expression libraries. In brief, organoids were transferred to HBSS (without Ca^2+^ or Mg^2+^, −/−) and cut into two pieces to clear away debris from the center of the organoid (two to three washes in total). Organoid pieces were then dissociated using the Neural Dissociation kit (P) and papain-based dissociation (Miltenyi Biotec). Organoid pieces were incubated in papain at 37 °C (enzyme mix 1) for an initial 15 min, followed by addition of enzyme A (enzyme mix 2) to the papain mix. Organoid pieces were then triturated using wide-bore 1,000-ml tips and incubated for additional intervals of 5–10 min with triturations between incubation steps, amounting to a total papain incubation time of approximately 45 min. Cells were filtered through a 30-μm strainer and washed, centrifuged for 5 min at 300*g* and washed three times with HBSS (−/−). Cells were filtered through a 20-μm strainer and washed, centrifuged for 5 min at 300*g* and washed three times with HBSS (−/−). The resulting cells were then assessed (count and viability) using the Trypan blue assay and counted using the automated cell counter Countess (Thermo Fisher). Finally, cells were diluted to an appropriate concentration to obtain approximately 5,000–7,000 cells per lane in a 10x microfluidic chip device. Single-cell cDNA was synthesized according to the manufacturer’s recommendations before continuing to library preparation with 25% of the total cDNA volume. Libraries were sequenced on the Illumina NovaSeq S1 and on the Illumina HiSeq 2500.

### Barcode and scar detection from single-cell cDNA

Barcode and scar regions were amplified from 60–70 ng of cDNA remaining from the scRNA-seq preparation with three separate PCR reactions. First, cDNA was amplified via PCR broadly targeting a region containing both the scar and the barcode. Subsequently, the reaction was split equally, and we performed a nested PCR separately targeting the barcode and scar regions (primer sequences are provided in Supplementary Table 5). Lastly, we added Illumina sequencing adaptors. Following every PCR reaction, samples were cleaned up using magnetic beads (0.9×) (Beckman Coulter), and libraries were sequenced on the Illumina NovaSeq S1.

### Alignment of single-cell transcriptomes and iTracer readouts

We used Cell Ranger (10x Genomics) to demultiplex base-call files to FASTQ files and align reads. Default alignment parameters were used to align reads to a modified human reference including the fluorescent reporter (GFP or RFP) from the cell lineage recorder (hg38). Barcode and scar libraries generated from 10x cDNA were also aligned using default parameters, with the exception that the force-cells argument was set to 200,000, and data were aligned to a custom reference of the region of interest. This reference was constructed following 10x recommendations.

### iTracer readout filtering

iTracer barcode transcripts were first filtered at the UMI level, at which transcripts are only retained if they have more than three reads. We then plotted the distribution of reads per UMI against the frequency of read depth per UMI and fit a line with loess through that distribution (loess (*n*_reads_ ≈ log (freq_*n*_reads_))), where values smaller than one were set to one. We then calculated the first minimum and removed everything that had smaller coverage than this point. iTracer barcodes began and ended with A or T nucleotides; barcodes that did not match this pattern were removed. We filtered barcode transcripts such that, when we detected the same UMI for different barcodes in the same cell, the one with the highest read coverage was retained. Furthermore, when transcripts with the same UMI and barcode were found in multiple cells, transcripts were removed from these cells. Barcodes were further filtered such that, when any barcodes with a Hamming distance of one within any single cell were found, the barcode with the highest coverage was retained. Lastly, if more than ten barcodes were detected in the same cell, the cell was ignored for lineage reconstructions.

iTracer scar transcripts were filtered in a similar manner, in which transcripts were first filtered at the UMI level; again, we only retained transcripts that had more than three reads. As we did for barcodes, we plotted the distribution of reads per UMI against the frequency of read depth per UMI and fit a line with loess through that distribution where values smaller than one were set to one. We calculated the first minimum and removed everything that had smaller coverage than this point. We also filtered transcripts such that, when we detected the same UMI for different scars in the same cell, the one with the highest read coverage was retained. Transcripts were further filtered out if they had the same UMI and scar but were found in multiple cells. Lastly, we only kept scar transcripts for which the same UMI was found in barcode and scar libraries. Similarly, barcode transcripts without corresponding scar transcripts were also excluded.

### Estimation of barcode complexity in the iTracer^+^ iCRISPR iPSC line

Single-cell iTracer barcode transcript readouts of three batches of the iTracer^+^ iCRISPR iPSC line were generated. There were 6,966, 5,316 and 4,253 cells detected in the three libraries, respectively, with, on average, 13,308 (13,218, 12,351 and 14,354) barcodes detected. For each library, on average, 62% ± 1% of detected barcodes were unique. As detected barcode numbers of the three batches were comparable, we simplified the scenario to three random samplings with the same sampling ratio (*p*) from the total population of *N* barcodes, where the likelihoods of different barcodes being selected are assumed to be equal. Therefore, the expected proportion of unique barcodes in one batch is (1 − *p*)^2^. The sampling ratio was therefore estimated to be ~21%, which suggests that the total number of barcodes available in the iTracer^+^ iCRISPR iPSC pool is roughly 60,000 or, more precisely, 62,597 based on the above calculation.

### Analysis of whole-organoid single-cell RNA-seq data

Seurat (version 3.1)^49^ was applied to scRNA-seq data for preprocessing. Ribosomal protein genes and pseudogenes were excluded from analysis. Generally, cells with more than 6,000 or less than 600 detected genes as well as those with a mitochondrial transcript proportion greater than 20% were excluded (Supplementary Table 1). After log normalization, 5,000 highly variable genes were identified using the default vst method, in which cell cycle-related genes were excluded (Supplementary Table 6). Cell cycle scores were then calculated and regressed out from highly variable gene expression to reduce its confounding effect. Regressed-out expression levels were then *z* transformed, followed by principal-component analysis (PCA) for dimension reduction. UMAP was applied to the top 20 principal components (PCs) for visualization.

To integrate data from different organoids, CSS^31^ was calculated as described. In brief, cells from each organoid were subset, and Louvain clustering (with a resolution of 0.6), implemented in Seurat, was applied based on the precalculated top 20 PCs. Average expression of the predefined highly variable genes was calculated for each cluster in each organoid. Afterward, Spearman correlation coefficients were calculated between every cell and every cluster in all organoids. For each cell, its correlations with different clusters of each organoid were *z* transformed. Its *z*-transformed similarities with clusters of different organoids were then concatenated as the final CSS representation. UMAP and Louvain clustering (with a resolution of 1) were applied to the CSS representation. Cluster annotation was carried out by combining expression patterns of canonical cell type markers, for example, *NES*, *DCX*, *SIX6*, *AIF1*, *DCN* and *EPCAM*, and VoxHunt^5,24^ to compare the average transcriptome of clusters to that of different mouse brain regions.

For each organoid, a four-layer lineage tree was reconstructed. The pseudo-root node, representing the organoid, was considered as the first layer. Barcode families, that is, cells with the same barcode combination detected, were considered as the second layer. Cells in the same barcode family were likely expanded from the same iPSC. In each barcode family, scar families, that is, cells with the same scar combination, were considered as the third layer, which represents cells in the organoid expanded from the same cell when Cas9 was induced. At the end, cells were considered as the fourth layer. Lineage trees were visualized with the ‘radialNetwork’ function in the ‘networkD3’ R package.

### Quantification of barcode family-composition similarity between clusters

Barcode family-composition similarity between two cell clusters was quantified as the number of cell pairs, with each cell in one cluster, that are of the same barcode family (denoted as *n*_*i*,*j*_; Fig. 3a). To control for confounding factors including cell numbers in clusters and organoid composition, 100 random shufflings of barcode family information for cells in each organoid were applied, and random composition similarity between two clusters was estimated in the same manner (denoted as *n*′_*i*,*j*_). The observed barcode family-composition similarity was then normalized into the *z* score $z_{i,j} = \left( {n_{i,j} - {\textrm{mean}}\left( {n\prime _{i,j}} \right)} \right)({\textrm{s.d.}}\left( {n\prime _{i,j}} \right))^{-1}$. *z* transformation was further applied to scale the resulting *z* scores between different cluster pairs (denoted as $\underline {z_{i,j}}$), and two cutoffs (0.01 and 0.99 quantiles of the standard normal distribution, that is, *z*_cutoff−_ = −2.33 and *z*_cutoff+_ = 2.33) were applied to obtain cluster pairs with significantly similar ($\underline {z_{i,j}} > z_{\textrm{cutoff} + }$) or different $\left( {\underline {z_{i,j}} < z_{\textrm{cutoff} - }} \right)$ barcode family composition. To identify groups of cell clusters with similar barcode family composition, hierarchical clustering was applied, with the input distance matrix defined as $d_{i,j} = {\textrm{max}}(z_{i,j}) - z_{i,j}$.

Alternatively, hierarchical clustering was applied to the binomial-based normalized barcode family-composition similarity distance matrix, which only takes into account the sizes of cell clusters. In brief, assuming the total number of cell pairs from the same barcode family is *N* and two clusters *i* and *j* represent proportions *p*_*i*_ and *p*_*j*_ of the whole dataset, the expected number of cell pairs in these two different clusters from the same barcode family is $\underline {n_{i,j}} = N \times 2p_ip_j$ with the expected standard deviation of $\sigma _{i,j} = \sqrt {N \times 2p_ip_j\left( {1 - 2p_ip_j} \right)}$, based on binomial distribution. The observed barcode family-composition similarity was then normalized into the alternative *z* score $z_{i,j} = \left( {n_{i,j} - n_{i,j}} \right)\sigma _{i,j}^{-1}$. Hierarchical clustering was then applied to identify groups of cell clusters with similar barcode family composition, with the input distance matrix defined as $d_{i,j} = {\textrm{max}}(z_{i,j}) - z_{i,j}$ with the alternative *z* scores.

### Spatial transcriptomics

Org14 was embedded in prechilled optimal cutting temperature compound. The sample was then set into a dry ice bath with isopentane until frozen and stored at −80 °C. Cryosections were cut at a thickness of 10 µm, adhered to ST slides (10x) and stored at −80 °C until the following day. Tissue slices were fixed in cold methanol before being stained with hematoxylin and eosin. ST slides were imaged as recommended on a Nikon T2i at 20× using a tile scan over all slice sections. Following image capture, tissue slices were permeabilized. Optimal permeabilization conditions were determined by using the Tissue Optimization kit (10x), and the optimal time was found to be 24 min. Spot-captured RNA was reverse transcribed before second-strand synthesis and cDNA denaturation. qPCR was used to determine the optimal number of cDNA-amplification cycles as recommended by the manufacturer. cDNA was amplified using 17–18 cycles before continuing to Visium spatial gene expression library construction. Visium libraries were sequenced on the Illumina NovaSeq SP following sequencing recommendations. Barcode and scar libraries were sequenced on the NextSeq (mid-output).

The resulting sequencing reads were aligned using Space Ranger for the regular Visium libraries (10x Genomics). S1 and S3 were automatically tissue aligned, whereas we manually annotated tissue-covering spots with Loupe Browser (10x Genomics) for S3. Spots not covering tissue were discarded manually in Loupe Browser (10x Genomics). Barcodes and scars were called using the methods described above with one exception. To use Cell Ranger to map barcode and scar sequencing reads to the custom reference, the Cell Ranger barcode whitelist was replaced with the whitelist from the Space Ranger barcode set.

Two methods were used to annotate spots. First, an elastic net-based machine learning classifier (implemented as glmnet in R) was trained by a random subset of whole-organoid scRNA-seq data with 2,626 cells in total, with each of the eight regional identities (telencephalic, dien–mesencephalic, rhombencephalic, retinal, neural crest derivatives, microglia, mesenchymal, epithelial) having no more than 400 cells, given highly variable gene expression as the input to predict cluster labels. The trained model was applied to transcriptomic profiles of spots to estimate the likelihood of each spot being different clusters. Likelihoods of clusters assigned to the same regional (or NPC–neuron state) identity were summed up, and spots were annotated to be the region or state with maximal likelihood, if it was at least 5% higher than the second largest estimated likelihood; otherwise, it was annotated as ‘unassigned’.

Spots were also annotated using CIBERSORTx^35^, with which we digitally sorted each spatial spot into fractions of cell types present. To this end, we first constructed a signature matrix for deconvolution using the highly variable genes and a subset of cells across all cell annotations from the whole-organoid analysis (Supplementary Table 7). We then input each detected spot across all tissue sections (S1–S3) for sorting, which resulted in a matrix of spots versus each cell annotation in which each row summed to 1. We plotted the distribution of the highest proportion (score) for each spot and set a threshold such that all spots with the highest contributing proportion less than the first quartile (0.405 or 40.5%) were called ‘unassigned’. The remaining spots were then assigned the corresponding cell annotation of their highest contributing proportion.

To quantify the relationship between barcode-composition differences and spatial proximity between spots, we first defined barcode-composition similarity between any two detected spots *i* and *j* as the Jaccard index (*J*_*i*,*j*_) of detected barcodes in the two spots, that is, the ratio of shared barcode number to unique barcode number. Barcode-composition distance was then defined as *d*_*i*,*j*_ = 1 − *J*_*i*,*j*_. The correlation between barcode-composition distances and spatial distances was next calculated. Alternatively, spot pairs were grouped into two groups: (1) spot pairs with at least one barcode shared and (2) spot pairs with no overlapping detected barcode. Spatial distances between spot pairs in the two groups were compared using two-sided Wilcoxon’s rank-sum test.

### Light-sheet imaging and tracking of cerebral organoids

We generated organoids using iPSCs expressing the FUS protein tagged with EGFP, which uniformly labels nuclei, and mTagRFP–T–CAAX, which labels membrane, and unlabeled iPSCs. FUS-mEGFP (cell line ID AICS-0080 cl.69), mTagRFP-T-CAAX (cell line ID AICS-0054-091) and WTC lines (cell line ID GM25256) used for imaging were procured from the Coriell Institute. Organoids were imaged with the LS1 Live light-sheet microscope developed by Viventis Microscopy, using a ×25 objective demagnified to ×18.5, with a field of view that was ~700 µm. Successive *z* steps were acquired every 2 µm for 150 steps. The frame rate for acquisition was 30 min, and, in total, 100 h of development (200 frames) were used for tracking. For imaging, EBs were embedded in neural-induction medium together with Matrigel. Light-sheet data were converted into HDF5 format and visualized using the BigDataViewer^50^ in Fiji^37^. In total, four nuclei were tracked, the first one for 100 h and the next three for 65 h. Three neighboring nuclei were tracked in the same developing lumen area and one in another diametrically opposite location surrounding another lumen in Org15. Nuclei were continually tracked in 3D using a new large-scale tracking and track-editing framework, Mastodon (preview), a next-generation software of the successful tools^51,52^ developed by the Tomancak laboratory at the MPI-CBG (https://sites.imagej.net/Mastodonpreview/) as a plugin in Fiji. It allows semi-automated tracking and manual curation of nuclei tracks. During lumen expansion and growth, some nuclei tracks are prematurely terminated when they move out of focus.

### Quantification of scar family-composition differences between clusters

To quantify scar family-composition differences between clusters, proportions of cells in different scar families of each barcode family were calculated. To increase the robustness of the estimate, one pseudocount was added to each scar family before calculating proportions. Here, only barcode families satisfying the following criteria were considered: (1) families that contain at least five scarred cells and (2) the second most frequent scar contains at least 10% of scarred cells in the barcode family. For each cell cluster, its scar family proportions of different barcode families in different organoids were concatenated to represent its scar family composition (denoted as *s*_*i*_). Scar family-composition differences between two clusters *i* and *j* (denoted as *d*_*i*,*j*_) were then defined as the Euclidean distance between *s*_*i*_ and *s*_*j*_.

To estimate the statistical significance of the scar family distance between two clusters, 1,000 random shufflings of scars were performed. During each shuffling, the scar information of cells in the same barcode family was randomly shuffled. Afterward, the shuffled scar family distance *d*_*i*,*j*_ was calculated in the same manner as described above. The observed scar family distance was then normalized into the *z* score $z_{i,j} = \left( {d_{i,j} - {\textrm{mean}}(d\prime _{i,j})} \right)({\textrm{s.d.}}\left( {d\prime _{i,j}} \right))^{-1}$. *A z* score that is significantly larger than zero indicates a significantly large scar family-composition difference between two clusters, implying that fate restriction occurred when scarring was induced.

To capture the global restriction signal, the distribution of observed *z* scores across all cluster pairs was subtracted by the average distribution of the 1,000 shuffling-based results, resulting in the excess of frequency at different *z* scores. If significant excess of frequency is observed for positive *z* scores, it indicates that more cluster pairs show significantly different scar family composition than expected by random chance, therefore implying that significant cell fate restriction occurred when scars were induced.

To identify cell clusters sharing similar scar family composition, hierarchical clustering was applied to the cell clusters of interest. The input distance matrix was defined as $D_{i,j} = z_{i,j} - {\textrm{min}}(z_{i,j})$.

### Regional heterogeneity in early cerebral organoid development

Four early cerebral organoids, which were cultured for 15 d with scars introduced at day 7, were dissociated to generate single-cell gene expression libraries. Dissociated cell suspensions were pooled into two samples, each representing two early organoids. Single-cell transcriptomes and iTracer readouts were preprocessed and filtered as described above.

Seurat (version 3.1) was applied to the transcriptome data for log normalization and highly variable gene identification (vst method, 3,000 genes). PCA was applied to the *z*-transformed expression levels of the highly variable genes, with the top 20 PCs used for UMAP embedding construction and Louvain clustering (with a resolution of 0.6). Cell clusters were annotated as pluripotent stem cells (PSCs), NECs, neural crest cells, mesodermal cells, endodermal cells and others based on expression of canonical markers (Extended Data Fig. 8). Cells annotated as PSCs and NECs were subset for the following analysis.

Focusing on the subset cells, highly variable genes were re-identified (vst, 3,000 genes). Genes related to the cell cycle were excluded from the identified genes. Cell cycle scores were calculated for cells following the vignette in Seurat and regressed out from the expression levels before applying *z* transformation of highly variable gene expression levels. PCA was then applied to the *z*-transformed expression levels, with the top 20 PCs used for UMAP embedding construction and Louvain clustering (with a resolution of 0.6). Cells were annotated as either PSCs or NECs.

To further annotate regional identities of cells in early cerebral organoids, scRNA-seq data of early neural tube cells and radial glia in the murine early fetal brain were retrieved by subsetting cells in the developing mouse brain scRNA-seq atlas^38^, requiring cells from samples no older than E11.0, annotated as neural tube cells and radial glia and with at least 3,000 genes detected. Seurat (version 3.1) was applied to identify highly variable genes (vst, 3,000 genes), and PCA of their *z*-transformed expression levels was performed. CSS^31^ was used to integrate and represent cells from different samples (default parameters). The CSS representation was used for UMAP embedding construction and Louvain clustering (with a resolution of 2). Cell clusters were annotated by combining sample meta-information of dissected tissues and regional markers. Regional identities of clusters (combinations of forebrain, midbrain and hindbrain) were determined by combining cluster annotation and proportions of cells from different dissected brain regions. High-resolution Louvain clustering (with a resolution of 20) was also applied to identify cell niches, with regional identities of niches summarized as the majority.

To project early cerebral organoid NECs to the atlas of mouse early neural tube cells and radial glia, we calculated the projected CSS for organoid cells by calculating normalized similarities between the cell transcriptome and the transcriptome of clusters in mouse reference samples^31^ across the one-to-one orthologous highly variable genes between human and mouse (Ensembl version 93) in the reference dataset. *k*-nearest neighbors (*k* = 50) of each organoid cell in the mouse reference were identified, and the major reference cell niche label was transferred to the organoid cell. The regional label of an NEC in the early cerebral organoid was then decided in a hierarchical manner. In brief, an NEC was defined as a hindbrain cell if the projected niche had hindbrain identity. Otherwise, it was defined as a midbrain cell if the projected niche had midbrain identity. Only if the projected niche only had forebrain identity, the NEC was defined as a forebrain cell.

Focusing on the 13 barcode families (six and seven barcode families in each of the two samples) with at least 20 PSCs and NECs, a two-sided *χ*^2^ test was applied to compare proportions of NECs with different estimated regional identities in different barcode families. On the other hand, a two-sided Fisher’s exact test was applied to cells in one barcode family (BF1 in sample 1) to compare proportions of NECs with different estimated regional identities in different scar families.

### Microdissection of single organoid regions

A 200-µm slice of a single organoid (Org13) was cut with a vibratome. Regions that were spatially distinct were selected and microdissected away from the slice. Each microdissected area was dissociated (as described above), adjusting times and volumes to account for smaller tissue input. Following single-cell isolation, cells were captured using 10x Chromium targeting 17,000 cells per region across four separate reactions such that each region was split into two capture reactions. Transcriptome, barcode and scar libraries were prepared as described above. All libraries were pooled and sequenced on the Illumina NovaSeq S1.

The resulting sequencing reads were aligned and preprocessed as described above. Seurat (version 3.1) was applied for log normalization and highly variable gene identification (vst method, 5,000 genes; Supplementary Table 6). PCA was applied to *z*-transformed expression levels of the highly variable genes, with the top 20 PCs used for UMAP embedding construction and Louvain clustering (with a resolution of 0.6). Cell cluster annotation was performed by combining canonical marker gene expression and CSS projection of cells to the whole-organoid scRNA-seq data described above. In brief, whole-organoid CSS representations of cells in the scRNA-seq data from microdissected samples were calculated as the normalized Spearman correlation between the cell transcriptome and the average transcriptome of cell clusters in whole-organoid scRNA-seq data. The *k*-nearest neighbors (*k* = 50) of each cell in the whole-organoid scRNA-seq data were identified as those with the shortest Euclidean distances at CSS representations. The major cell cluster label of the identified neighbors was assigned to the query cell in the scRNA-seq data from microdissected samples as the transferred label to assist annotation of scRNA-seq data from microdissected samples.

Barcode family-composition similarities between cell clusters were quantified as described above. Hierarchical clustering was applied to the random barcode shuffling-based quantification (ward.D2 method) to group cell clusters into three groups. Scar family-composition differences between cell clusters in each of the three groups were then quantified as described above.

To study the neurogenic synchrony of different stem cells, pseudotime analysis was applied to cells from the microdissected R2, which were in CG3-1. Pseudotime analysis was performed with a diffusion map (implemented in the destiny R package) on the top 20 PCs of selected cells. The ranked first diffusion component was used as the pseudotime value of each cell. The constructed pseudotime course was split evenly into ten bins. The number of cells in each scar family and each barcode family was counted for each pseudotime bin. Distributions of cells in different pseudotime bins of different barcode or scar families were compared using a *χ*^2^ test.

### Establishment of iTracer-perturb

iTracer-perturb plasmids were constructed by modifiying the iTracer plasmids in four main steps. All steps were carried out using Gibson assembly unless otherwise specified. (1) Replacement of the sequence for GFP on iTracer with that for RFP–2A–GFP or BFP–2A–GFP. (2) Cloning and incorporation of *TSC2*-targeting or nontargeting gRNA species driven by the bovine U6 promoter. (3) Construction of the pLenti vector containing a hygromycin resistance gene driven by the *PGK* promoter. (4) Movement of iTracer-perturb from the pSBbi backbone to the pLenti backbone. In detail, the sequence for the 2A peptide was included as an overhang on oligonucleotides used to amplify sequences for GFP, RFP and BFP. The overhand was turned into an overlap region that enabled assembly onto the long-range PCR-amplified iTracer backbone. Sequences for either *TSC2*-targeting or nontargeting gRNA were cloned into the PMJ114 vector (Addgene, 85995) downstream of the bovine U6 promoter following instructions in the Perturb-seq manual. Sequences for corresponding bovine U6 promoter-driven gRNA species were amplified by PCR and cloned upstream of sequences for human U6 promoter-driven gRNA species targeting *GFP* in the iTracer plasmid. In parallel, the *PGK* promoter and hygromycin resistance gene were respectively amplified from AAVS1-mEGFP (Addgene, 114404) and the Hygro-Cas9 donor (Addgene, 86883) and assembled onto the pLenti backbone from PMJ114. iTracer-perturb was then moved from the pSBbi backbone to the pLenti vector between the 5′-LTR and *PKG*-*hygroR*. Barcodes generated by PCR using oligonucleotides containing random nucleotides as a template were introduced at the last step into the pLenti-PLT vector.

Lentiviruses were packaged by first culturing HEK293T cells in DMEM with 10% FBS and antibiotics at 37 °C in an atmosphere with 5% CO_2_. To prepare lentiviruses, HEK293T cells were passaged 1:2 every day for at least 2 d for optimal cell conditions. Cells at 90% confluency were transfected with pLenti plasmids, pGag/pol, pVSVG, pRev and pTat using the TransIT-293 transfection reagent (Mirus Bio). Medium containing transfection reagents was replaced 16 h after transfection with rich medium containing 30% FBS. Lentiviruses were collected 48 h after transfection and concentrated 50× using the Lenti-X concentrator (Takara Bio) following the manufacturer’s instructions. Concentrated lentiviruses were preserved in a −80 °C freezer until use.

### Validation of TSC2 guide cutting

Guides were designed using CRISPRdirect targeting exons 11, 13, 22 and 28 of *TSC2* (ref. ^53^): GGCCATGGCATGTCCGAACG, GTGGATGGACTGCGCTCTAT, TGGACAAGGCCACGACGCAC and ACCGGGACCCGGTCGTTACT, respectively. iCRISPR iPSCs were cultured in StemFlex medium (Gibco, A3349401) supplemented with 1:200 penicillin–streptomycin (Gibco, 15140-122) unless otherwise mentioned. Before lipofection (24 h), cells were treated either without or with 2 µg ml^−1^ doxycycline (Clontech, 631311) in StemFlex medium to induce Cas9 expression. The Alt-R CRISPR–Cas9 System (IDT) was used for guide delivery with lipofection according to the manufacturer’s protocol. To form the crRNA–tracrRNA complex at a final concentration of 3 µM for each guide complex, 1.5 µl of each guide crRNA was combined with 3 µl tracrRNA and 44 µl Nuclease-Free Duplex Buffer (IDT, 11-01-03-01) and heated at 95 °C for 5 min. For reverse transfection, 1.5 µl of the crRNA–tracrRNA complex mix and 0.75 µl RNAiMAX (Invitrogen, 13778075) were diluted in 47.75 µl Opti-MEM (Gibco, 1985-062) and incubated for 20 min at room temperature. During incubation, ∼70% confluent iCRISPR cells from both the no-doxycycline control and doxycycline-exposed cells were detached separately with TrypLE (Gibco, 12605010), centrifuged and resuspended at a concentration of 400,000 cells per ml in StemFlex medium without penicillin–streptomycin supplemented with 1:10 CloneR (Stemcell, 05888). Additionally, 2 µg ml^−1^ doxycycline was added to the doxycycline-exposed cell suspension. In total, 100 µl of the cell solution and 50 µl of the lipofection solution were combined for each condition in a well of a 96-well plate coated with Matrigel (Corning, 35248). After 24 h, wells were washed with 150 µl DPBS (Gibco, 14190250), and medium was exchanged with 150 µl fresh StemFlex medium. After lipofection (48 h), cells reached ∼80% confluency. Wells were washed with 150 µl DPBS, and the gDNA content of cells was extracted by adding 50 µl QuickExtract solution (Epicentre, QE0905T) according to the manufacturer’s protocol. Amplification of each guide target site was performed by PCR using specific primers containing Illumina sequencing adaptors (Supplementary Table 5).

gDNA treated with QuickExtract (1 µl) from each condition was combined with 1 µl of the corresponding forward and reverse primers, 10 µl Phusion HF MasterMix (Thermo Scientific, F-531L) and 7 µl nuclease-free water before the reaction was run in a thermocycler with the following program: 98 °C, 30 s; 30 × (98 °C, 8 s; 62 °C, 20 s; 72 °C, 22 s); 72 °C, 7 min 30 s. Indexing PCR was then performed by combining 1 µl of the previous PCR product with 12.5 µl Phusion HF MasterMix, 9 µl nuclease-free water and 1.25 µl unique P5 and P7 Illumina indices from the Nextera XT Index kit version 2, set A (Illumina, FC-131-2001) before running the following thermocycler program: 98 °C, 30 s; 25 × (98 °C, 10 s; 58 °C, 10 s; 72 °C, 20 s); 72 °C, 5 min. Double-indexed libraries were pooled and purified twice with SPRI beads (0.9×). Purified libraries were sequenced on the MiSeq (Illumina), resulting in paired-end sequences of 2 × 150 bp. Finally, guide cutting efficiency was determined from the sequencing results using CRISPResso2 (ref. ^54^) with default settings.

### Preparation of iTracer-perturb organoids

iCRISPR cells were cultivated in six-well plates using standard feeder-free conditions in mTeSR1 (Stemcell Technologies) on Matrigel-coated plates. Cells were infected with 65 µl lentivirus per well. Transfected cells were allowed to expand for 4 d before flow cytometry. To select for cells with successful integration of both *TSC2*-targeted and nontargeted iTracer-perturb, RFP^+^GFP^+^ and BFP^+^GFP^+^ cells were sorted into separate flow cytometry tubes and plated in 12-cell Matrigel-coated plates with mTeSR1. Following cell recovery after flow cytometry, 2,000 cells per well in a 96-well plate (10% RFP^+^GFP^+^, 10% BFP^+^GFP^+^ and 80% wild-type cells) were seeded and differentiated into cerebral organoids using a whole-organoid differentiation protocol^5,22^. Organoids were scarred by activation of inducible Cas9 at day 5. Scarring was achieved using 8 µg ml^−1^ doxycycline in NIM for 24 h. Organoids were incubated in scarring medium for 24 h before returning to base medium without doxycycline.

Organoids were dissociated as previously described, and RFP^+^, GFP^+^ and BFP^+^ cells were sorted into separate pools by flow cytometry. Cell pools were directly loaded onto the 10x microfluidic chip device. Single-cell cDNA was synthesized according to the manufacturer’s recommendations before continuing to library preparation with 25% of the total cDNA volume. Libraries were sequenced on the Illumina NovaSeq S1. Barcode and scar regions were amplified from 30–50 ng of cDNA remaining from the scRNA-seq preparation with three separate PCR reactions. First, cDNA was amplified, broadly targeting a region containing both the scar and barcode; this was performed by monitoring qPCR amplification and stopping amplification before the plateau. Subsequently, the reaction was split equally, and we performed PCR, separately targeting the barcode and scar regions (primer sequences are provided in Supplementary Table 5). Lastly, we added Illumina sequencing adaptors. Following every PCR reaction, samples were cleaned up using magnetic beads (0.9×) (Beckman Coulter), and libraries were sequenced on the Illumina NovaSeq S1.

### Analysis of iTracer-perturb

scRNA-seq and iTracer readouts were aligned as previously described. Seurat (version 4) was applied to scRNA-seq data for preprocessing. Generally, cells with less than 1,000 detected genes as well as those with mitochondrial transcript proportion greater than 10% were excluded. After log normalization, 3,000 highly variable genes were identified using the default vst method, in which cell cycle-related genes, mitochondrial genes and ribosomal protein-encoding genes were excluded. Cell cycle scores were then calculated and regressed out from highly variable gene expression to reduce its confounding effect. Regressed-out expression levels were then *z* transformed, followed by PCA for dimension reduction (the top 20 PCs were used). CSS was then calculated in a manner similar to that described above (clustering resolution of 1) to integrate cells sorted by different fluorescence signals. PCA was then applied to further summarize the calculated CSS into ten PCs. UMAP and Louvain clustering (with a resolution of 0.5) was applied to the PCA-on-CSS representation. Cluster annotation was performed by combining expression patterns of canonical cell type markers. We detected at least one barcoded transcript for GFP in 221 RFP^+^ and 3,808 BFP^+^ cells, with 19.7% of these cells containing at least one GFP scar. For each cluster, the number of RFP^+^ or BFP^+^ cells with at least one GFP scar detected (that is, fully informative cells) was calculated. Cell clusters with at least 100 fully informative cells were defined as sufficiently scarred clusters and used for the following analysis. Mixscape analysis, adapted from the online tutorial (https://satijalab.org/seurat/articles/mixscape_vignette.html), was applied to cells in these clusters to obtain the posterior perturbation probability, using BFP^+^ cells as nontargeting cells and RFP^+^ cells as targeting cells. Next, a five-layer lineage tree was reconstructed for cells in the sufficiently scarred clusters using a method similar to the one described above. One extra layer (RFP^+^ or BFP^+^) was added between the organoid layer and the barcode family layer. UMAP and Louvain clustering (with a resolution of 1) was further applied to dissect cell state heterogeneity.

To compare transcriptomic profiles of RFP^+^ and BFP^+^ cells as well as different scar families of BFP^+^ cells, we applied analysis of covariance to genes detected in at least 1% of cells. For each gene, we applied an F-test to compare the full model with both covariates (cell clusters and log-transformed UMI numbers) and the condition variable (for example, RFP^+^ or BFP^+^) and the reduced model with only the covariates. DEGs were defined as genes with Bonferroni-corrected *P* < 0.01, with the coefficient of the condition variable greater than 0.1 or less than −0.1.

To compare the cell state trajectory of different cell lineages, pseudotime was constructed to represent the cell state transition from NECs to NPCs. In brief, cell cycle scores were first regressed out from PCA-on-CSS representations, followed by diffusion map analysis of the resulting representations. The resulting first diffusion component was ranked across cells and considered as the pseudotime.

### Flow cytometry

To select iTracer^+^ iPSCs, cells were detached from Matrigel-coated plates to create a single-cell suspension. Briefly, cells were washed with DPBS before being lifted from the Matrigel plate with Accutase for 5–7 min before quenching with KnockOut Media and centrifuging at 200*g* for 5 min. Cells were resuspended and filtered through a 30-µm cell strainer in 500 µl mTeSR1 supplemented with Rock inhibitor (1:200) and Primocin (1:500). Cells were sorted on the BD Aria Fusion or the BD Aria III using a 100-µm nozzle, selecting for single cells and excluding doublets, based on their florescent reporter. For iTracer experiments, selection was based on GFP or RFP positivity, whereas iTracer-perturb cells were identified based on dual expression (RFP and GFP; BFP and GFP). All cells were sorted into mTeSR1 medium supplemented with PenStrep (1:200), Rock inhibitor (1:200) and Primocin (1:500). Flow cytometry data were visualized with BD FACSDiva 9.0.1.

To select iTracer-perturb cells from organoids, organoids were first dissociated as previously described. After dissociation, cells were resuspended in DPBS supplemented with BSA (0.04%, Miltenyi, 130-091-376). Cells were sorted on the BD Aria Fusion or the BD Aria III using a 100-µm nozzle, selecting for single cells and excluding doublets, based on their florescent reporter (dual BFP- and GFP-, dual RFP- and GFP- or GFP-positivity alone). All cells were sorted into DPBS supplemented with BSA (0.04%), and flow cytometry data were visualized with BD FACSDiva 8.1.

### Statistics and reproducibility

No statistical method was used to predetermine sample size. No data were excluded from analysis. Experiments were not randomized. Investigators were not blinded to allocation during experiments and outcome assessment. Boxes in all the box plots represent upper and lower quartiles. The center line represents the median. Whiskers show the minimum and maximum of the data if there is no outlier. Outliers are defined as data points outside 1.5 times the interquartile range above the upper quartile and below the lower quartile. Only one cerebral organoid was imaged by 4D light-sheet microscopy and is shown in Fig. 3b–d and Extended Data Fig. 6a,c. Bright-field and fluorescence imaging of iTracer reporter (RFP) in organoids shown in Extended Data Fig. 1b is representative of images of multiple organoids (*n* > 10).

### Reporting Summary

Further information on research design is available in the Nature Research Reporting Summary linked to this article.

## Online content

Any methods, additional references, Nature Research reporting summaries, source data, extended data, supplementary information, acknowledgements, peer review information; details of author contributions and competing interests; and statements of data and code availability are available at 10.1038/s41592-021-01344-8.

## Supplementary information

Reporting Summary

Supplementary Video 1

Supplementary Video 2

Supplementary Video 3

Supplementary Video 4

Supplementary Table 1

Supplementary Table 2

Supplementary Table 3

Supplementary Table 4

Supplementary Table 5

Supplementary Table 6

Supplementary Table 7

Supplementary Table 8

## Data Availability

Sequence data that support the findings of this study have been deposited in ArrayExpress under the accession codes E-MTAB-10973, E-MTAB-10974 (scRNA-seq data based on 10x Genomics), E-MTAB-10972 (spatial transcriptomic RNA-seq data based on 10x Visium) and E-MTAB-10971 (iTracer-perturb data). Bulk sequencing of barcode and scar library data as well as processed sequencing data have been deposited in Mendeley Data at 10.17632/nj3p3pxv6p.

## Code availability

The computational code used in this study is available at GitHub (https://github.com/quadbiolab/iTracer) or upon request.
