# Supplementary material for: Lineage recording in human cerebral organoids
Source: Nat Methods. 2021 Dec 30;19(1):90–9. doi: 10.1038/s41592-021-01344-8 (PMC8748197; doi:10.1038/s41592-021-01344-8)
Supplement: Supplementary file 1 — Reporting Summary [file 41592_2021_1344_MOESM1_ESM.pdf]

nature research

Corresponding author(s): Barbara Treutlein, J. Gray Camp

Last updated by author(s): Sep 27, 2021

# Reporting Summary

Nature Research wishes to improve the reproducibility of the work that we publish. This form provides structure for consistency and transparency in reporting. For further information on Nature Research policies, see our Editorial Policies and the Editorial Policy Checklist.

## Statistics

For all statistical analyses, confirm that the following items are present in the figure legend, table legend, main text, or Methods section.

| n/a | Confirmed | |
|---|---|---|
| ☐ | ☒ | The exact sample size (*n*) for each experimental group/condition, given as a discrete number and unit of measurement |
| ☐ | ☒ | A statement on whether measurements were taken from distinct samples or whether the same sample was measured repeatedly |
| ☐ | ☒ | The statistical test(s) used AND whether they are one- or two-sided<br>*Only common tests should be described solely by name; describe more complex techniques in the Methods section.* |
| ☐ | ☒ | A description of all covariates tested |
| ☐ | ☒ | A description of any assumptions or corrections, such as tests of normality and adjustment for multiple comparisons |
| ☐ | ☒ | A full description of the statistical parameters including central tendency (e.g. means) or other basic estimates (e.g. regression coefficient) AND variation (e.g. standard deviation) or associated estimates of uncertainty (e.g. confidence intervals) |
| ☐ | ☒ | For null hypothesis testing, the test statistic (e.g. *F*, *t*, *r*) with confidence intervals, effect sizes, degrees of freedom and *P* value noted<br>*Give P values as exact values whenever suitable.* |
| ☒ | ☐ | For Bayesian analysis, information on the choice of priors and Markov chain Monte Carlo settings |
| ☒ | ☐ | For hierarchical and complex designs, identification of the appropriate level for tests and full reporting of outcomes |
| ☒ | ☐ | Estimates of effect sizes (e.g. Cohen's *d*, Pearson's *r*), indicating how they were calculated |

*Our web collection on statistics for biologists contains articles on many of the points above.*

## Software and code

Policy information about availability of computer code

| | |
|---|---|
| Data collection | See methods p. 20-26, 28 |
| Data analysis | See methods p. 19-25, 29 |

For manuscripts utilizing custom algorithms or software that are central to the research but not yet described in published literature, software must be made available to editors and reviewers. We strongly encourage code deposition in a community repository (e.g. GitHub). See the Nature Research guidelines for submitting code & software for further information.

## Data

Policy information about availability of data

All manuscripts must include a data availability statement. This statement should provide the following information, where applicable:

- Accession codes, unique identifiers, or web links for publicly available datasets
- A list of figures that have associated raw data
- A description of any restrictions on data availability

Sequence data that support the findings of this study have been deposited in ArrayExpress with the accession codes E-MTAB-10973, E-MTAB-10974 (single-cell RNA-seq data based on 10x Genomics), E-MTAB-10972 (spatial transcriptomics RNA-seq data based on 10x Visium) and E-MTAB-10971 (iTracer-perturb data). Processed sequencing data have been deposited in Mendeley Data with doi http://doi.org/10.17632/nj3p3pxv6p

# Field-specific reporting

Please select the one below that is the best fit for your research. If you are not sure, read the appropriate sections before making your selection.

☒ Life sciences ☐ Behavioural & social sciences ☐ Ecological, evolutionary & environmental sciences

For a reference copy of the document with all sections, see nature.com/documents/nr-reporting-summary-flat.pdf

# Life sciences study design

All studies must disclose on these points even when the disclosure is negative.

| | |
|---|---|
| Sample size | For determining the number of cells to be sequenced per organoid, the analysis done in our project is as extensive as any previous study; based on Kanton et al. (Nature 2019) and Velasco et al. (Nature, 2019), we believe that we sufficiently sample the heterogeneity with the number of cells sequenced. |
| Data exclusions | We excluded low quality cells using criteria as described in the Methods (p. 21). We excluded one tissue section from the spatial transcriptomics experiment due to misalignment on the capture area. Beyond this, no other data were excluded. |
| Replication | We analyzed multiple organoids to determine the reproducibility of gene expression and lineage recording patterns across organoids. We analyzed multiple organoids for different scarring time points. |
| Randomization | All organoids were selected at random from organoid batches. |
| Blinding | Investigators were not blinded during data acquisition. |

# Reporting for specific materials, systems and methods

We require information from authors about some types of materials, experimental systems and methods used in many studies. Here, indicate whether each material, system or method listed is relevant to your study. If you are not sure if a list item applies to your research, read the appropriate section before selecting a response.

## Materials & experimental systems

| n/a | Involved in the study | |
|---|---|---|
| ☒ | ☐ | Antibodies |
| ☐ | ☒ | Eukaryotic cell lines |
| ☒ | ☐ | Palaeontology and archaeology |
| ☒ | ☐ | Animals and other organisms |
| ☒ | ☐ | Human research participants |
| ☒ | ☐ | Clinical data |
| ☒ | ☐ | Dual use research of concern |

## Methods

| n/a | Involved in the study | |
|---|---|---|
| ☒ | ☐ | ChIP-seq |
| ☐ | ☒ | Flow cytometry |
| ☒ | ☐ | MRI-based neuroimaging |

## Eukaryotic cell lines

Policy information about cell lines

| | |
|---|---|
| Cell line source(s) | See methods p. 17 (iCRISPR cell line) and p. 23 (Lightsheet imaging and tracking of cerebral organoid) |
| Authentication | See methods p. 17 |
| Mycoplasma contamination | Cell lines were tested for mycoplasma contamination on a regular basis using a PCR-based test and were found to be negative for mycoplasma. |
| Commonly misidentified lines (See ICLAC register) | None. |

## Flow Cytometry

### Plots

Confirm that:

☒ The axis labels state the marker and fluorochrome used (e.g. CD4-FITC).

☒ The axis scales are clearly visible. Include numbers along axes only for bottom left plot of group (a 'group' is an analysis of identical markers).

☐ All plots are contour plots with outliers or pseudocolor plots.

☐ A numerical value for number of cells or percentage (with statistics) is provided.

### Methodology

| | |
|---|---|
| Sample preparation | Cells were brought into suspension with Accutase and incubation for 5 minutes at 37C. Knock Out media was used to quench the reaction before cells were spun and supernatant was removed. The cell pellet was resuspended in FACS media (mTSER1 media, 1:500 Rock Inhibitor, and 1:500 Primocin), and filtered through a 30um mesh filter into a FACS tube for sorting. |
| Instrument | BD FACS Aria Fusion and FACSAria III |
| Software | BD FACSAria™ software |
| Cell population abundance | The final sorted population was 0.1-8% of the total events. |
| Gating strategy | For iTracer experiments: three gates were used 1) FSC-A vs SSC was used to gate for the bulk population of cells, 2) FSC-A vs FSH-H or SSC-A vs SSC-H was used to minimize doublet sorting, 3) GFP+ or RFP+ populations were determined and gated for comparing to GFP/RFP negative controls.<br>For iTracer-perturb experiments: four gates were used to sort for iTracer-perturb+ iPSCs 1) FSC-A vs SSC was used to gate for the bulk population of cells, 2) FSC-A vs FSH-H or SSC-A vs SSC-H was used to minimize doublet sorting, 3) GFP+/RFP+ or 4) GFP+/BFP+ populations were gated and sorted as determined by comparing to negative control populations. Four gates were used to sort for iTracer-perturb+ cells from organoids 1) FSC-A vs SSC was used to gate for the bulk population of cells, 2) FSC-A vs FSH-H or SSC-A vs SSC-H was used to minimize doublet sorting, 3) RFP+ or 4) BFP+ populations were gated and sorted as determined by comparing to internal wild type control populations. |

☒ Tick this box to confirm that a figure exemplifying the gating strategy is provided in the Supplementary Information.
